# Genome-Wide Identification of the Two-Pore Potassium Channel Gene Family in Sugar Beet (*Beta vulgaris* L.) and Functional Analysis of *BvTPK2* in Salt Tolerance

**DOI:** 10.3390/plants15203156

**Published:** 2026-10-15

**Authors:** Shuai Wang, Yuzhu Cui, Yue Li, Yuxin Wang, Yaqing Sun, Guolong Li, Ningning Li, Shaoying Zhang

**Affiliations:** College of Agriculture, Inner Mongolia Agricultural University, Hohhot 010019, China; shuaiwang@email.imau.edu.cn (S.W.);

**Keywords:** sugar beet, two-pore K^+^ channels, *BvTPK2*, salt tolerance

## Abstract

Tandem-pore potassium (TPK/KCO) channels play important roles in plant salt tolerance and K^+^ homeostasis. However, the TPK/KCO gene family has not been systematically characterized in sugar beet (*Beta vulgaris* L.), the world’s second-largest sugar crop, and its functions in salt-stress responses remain largely unknown. In this study, six TPK/KCO family members were identified from the sugar beet genome and systematically analyzed by bioinformatics and expression analyses. The *BvTPK* genes were unevenly distributed across three chromosomes, designated *BvTPK1*–*BvTPK6*, and contained characteristic Ion_trans_2 and PRK10537 domains. Phylogenetic analysis classified TPK/KCO proteins into three major groups, with BvTPK1, BvTPK2, BvTPK3, and BvTPK6 in Group II, BvTPK5 in Group I, and BvTPK4 in Group III. The promoter regions of *BvTPKs* contained numerous cis-regulatory elements associated with light, hormone, and stress responses. Transcriptomic and RT–qPCR analyses revealed that several *BvTPK* genes responded to salt stress, with *BvTPK2* showing strong salt-induced expression in leaves. Subcellular localization confirmed that BvTPK2 was localized to the tonoplast. Moreover, heterologous overexpression of *BvTPK2* enhanced seed germination and survival, improved plant growth and fresh and dry weights, and maintained better photosynthetic performance in *Arabidopsis* under salt stress, indicating that *BvTPK2* positively contributes to salt tolerance. The overexpressing lines also exhibited reduced Na^+^ accumulation, enhanced K^+^ retention, and lower Na^+^/K^+^ ratios, indicating improved ionic homeostasis under salt stress. Collectively, these findings provide a characterization of the *TPK/KCO* gene family in sugar beet and identify *BvTPK2* as a key candidate gene associated with salt tolerance.

## 1. Introduction

Soil salinization is a major abiotic constraint on global agricultural production, severely limiting crop growth, productivity, and quality [1,2]. Therefore, elucidating the functions and regulatory mechanisms of genes involved in salt tolerance, together with the application of gene-editing and multi-trait pyramiding strategies, is crucial for the molecular breeding of salt-tolerant crops and the sustainable utilization of salt-affected soils [3]. Sugar beet (*Beta vulgaris* L.) is the world’s second most important sugar crop after sugarcane. Compared with major cereal crops such as rice and wheat, sugar beet exhibits relatively high salt tolerance and can maintain substantial storage-root yield and sugar content under moderately saline conditions, making it a promising crop for cultivation in salt-affected soils and marginal lands [4]. The identification and functional characterization of salt-tolerance genes in sugar beet will not only facilitate the development of salt-tolerant cultivars but also provide valuable genetic resources and mechanistic insights for improving salt tolerance in other crop species [5].

The tandem-pore potassium channel (TPK/KCO) family are plant-specific K^+^-selective channels characterized by four transmembrane helices and two tandem-pore-forming domains [6]. Members of this family have historically been designated as either TPK (tandem-pore potassium channel) or KCO (K^+^ channel with a consensus sequence) due to differences in gene discovery and annotation systems among plant species. Most TPK/KCO family members localize to the tonoplast, whereas some localize to the plasma membrane or other endomembrane compartments. Through their roles in K^+^ transport and intracellular compartmentalization, these channels regulate plant growth and responses to salinity, drought, and temperature stress [7,8]. *KCO1*, the first member of this family to be cloned, encodes an outward-rectifying K^+^ channel. The activity of the encoded channel is regulated by cytosolic Ca^2+^, thereby establishing a link between Ca^2+^ signaling and K^+^ transport [9]. The *Arabidopsis* TPK/KCO family comprises five tandem-pore TPK channels and one single-pore KCO channel. Except for AtTPK4, which is predominantly localized to the plasma membrane of pollen cells, the other members are mainly localized to the tonoplast [8,10]. *AtTPK1* is widely expressed in leaves, flowers, seedlings, and actively dividing tissues, while the AtTPK1 channel can be activated by Ca^2+^-dependent phosphorylation and contributes to vacuolar K^+^ homeostasis under salt stress [11]. TPK/KCO family members have subsequently been characterized in various plant species, although different naming conventions have been adopted. In rice, OsTPKa and OsTPKb are both localized to the tonoplast but are specifically targeted to lytic and protein storage vacuoles, respectively, through C-terminal sorting signals [12]. *OsTPKa* expression is induced by salt stress in salt-tolerant rice cultivars. Its downregulation aggravates salt-induced damage, whereas its overexpression promotes plant growth and enhances salt tolerance, indicating that *OsTPKa* positively regulates salt-stress adaptation in rice [13]. In soybean, nine members of the TPK/KCO family (GmTPK/KCO genes) have been identified among K^+^ transporter-related gene families [14]. In mango, 37 K^+^ transport-related genes have been identified, including six TPK genes. These genes exhibit distinct drought-responsive expression patterns in roots and leaves, and *MiTPK1.1* is differentially expressed during fruit development [15]. In wheat, TaTPK genes belonging to the TPK/KCO family have been identified; nine encode proteins predicted to localize to the plasma membrane, whereas the remaining three encode proteins predicted to localize to the tonoplast. Their expression levels are significantly altered by heat, drought, combined heat and drought, salt stress, and fungal infection, suggesting that these genes participate broadly in biotic and abiotic stress responses [16].

Functional evidence from other plant species further supports the involvement of TPK/KCO family members in stress adaptation. The PeTPK1 protein from *Populus euphratica* shares 81% sequence identity with AtTPK1, and overexpression of *PeTPK1* in tobacco BY-2 cells enhances salt tolerance. Under salt stress, *PeTPK1* facilitates K^+^ release from the vacuole into the cytosol, thereby contributing to intracellular K^+^ homeostasis [17]. In rice, overexpression of *OsTPKb* under salt stress increases the cytosolic-to-vacuolar K^+^ concentration ratio and thereby contributes to enhanced stress tolerance [18].

Nevertheless, the TPK/KCO family has not been systematically characterized in sugar beet, and the roles and regulatory mechanisms of its key members in salt tolerance remain largely unknown. In this study, we systematically identified and characterized the TPK/KCO family in sugar beet, with the identified members designated as *BvTPK1*–*BvTPK6* according to the commonly used TPK nomenclature, and investigated their expression patterns under salt stress. Based on these analyses, the salt-responsive candidate gene *BvTPK2* was selected for further characterization, including subcellular localization and functional evaluation of seed germination and survival in transgenic *Arabidopsis* under salt stress. These findings provide a basis for understanding the involvement of TPK/KCO family members in salt-stress responses of sugar beet and for further investigation of their functions in salt tolerance.

## 2. Results

### 2.1. Genome-Wide Identification Analysis of the TPK/KCO Family in Sugar Beet

**Figure 1:**
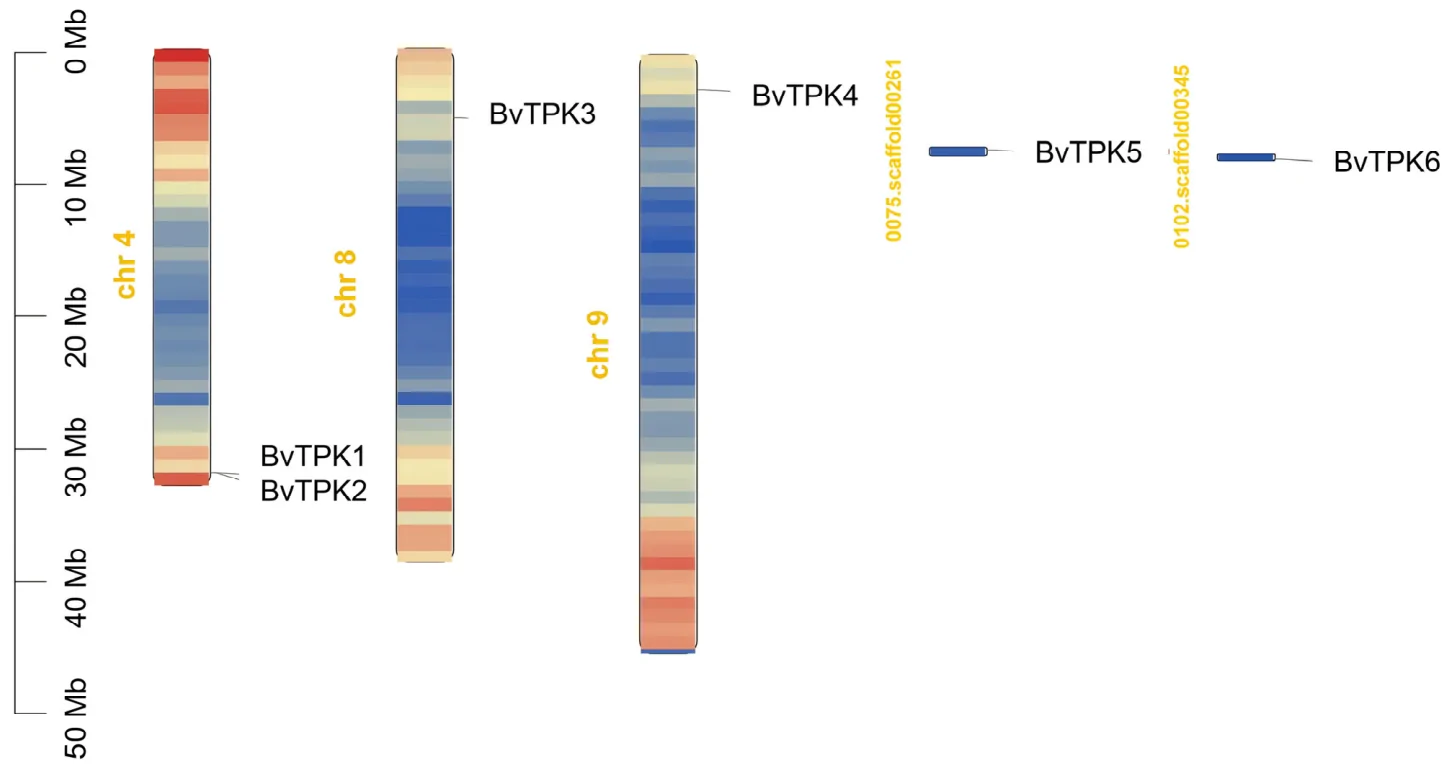
Chromosomal localization of the *TPK/KCO* family in sugar beet. Six TPK/KCO family members were unevenly distributed across the sugar beet genome. Two genes (*BvTPK1 and BvTPK2*) were located on chromosome 4, one gene (*BvTPK3*) on chromosome 8, and one gene (*BvTPK4*) on chromosome 9, while *BvTPK5* and *BvTPK6* were mapped to unanchored scaffolds. Chromosome lengths are shown in megabases (Mb). The colors along the chromosomes represent variations in gene density.

A total of six members of the TPK/KCO family were identified in the sugar beet genome through sequence screening and conserved domain verification. The candidate genes were identified based on a combination of sequence homology comparison, phylogenetic analysis, conserved domain analysis, and structural feature verification. SMART analysis was used to confirm the presence of conserved domains rather than as the sole criterion for family identification. Based on sequence homology, phylogenetic relationships, and the commonly used TPK nomenclature, these genes were designated as *BvTPK1*–*BvTPK6*. Multiple sequence alignment further revealed that all BvTPK proteins contained conserved regions characteristic of TPK/KCO family proteins, including the conserved pore-forming region and the GYGD selectivity filter motif (Supplementary Figure S2), supporting their classification as members of the TPK/KCO family. Chromosomal localization analysis revealed that the *BvTPK* genes were unevenly distributed across the sugar beet genome (Figure 1). Specifically, two genes (*BvTPK1*–*BvTPK2*) were located on chromosome 4, whereas *BvTPK3* and *BvTPK4* were mapped to chromosomes 8 and 9, respectively. The remaining two genes, *BvTPK5* and *BvTPK6*, were assigned to unanchored scaffolds and therefore could not be anchored to specific chromosomes. These results indicate a non-uniform chromosomal distribution pattern of the TPK/KCO family in sugar beet.

The physicochemical properties of the predicted BvTPK proteins are summarized in Table 1. Protein lengths ranged from 237 to 461 amino acids, with predicted molecular weights ranging from 26.65 to 51.48 kDa and theoretical isoelectric points ranging from 5.52 to 8.81. Instability index analysis predicted that BvTPK2 and BvTPK5 were unstable proteins (instability index > 40), whereas BvTPK6 was at the threshold value (40.00) and the remaining members had instability indices below 40. All BvTPK proteins exhibited aliphatic index values above 80, suggesting relatively high thermostability. Hydropathicity analysis showed that the GRAVY values of BvTPK proteins ranged from −0.126 to 0.435, with most members showing positive values, whereas BvTPK4 had a negative value. Subcellular localization prediction suggested that BvTPK proteins were distributed in diverse cellular compartments, including the plasma membrane, vacuole, and endoplasmic reticulum, indicating potential functional divergence among family members.

### 2.2. Phylogenetic and Synteny Analyses of the TPK/KCO Family in Sugar Beet and Related Species

**Figure 2:**
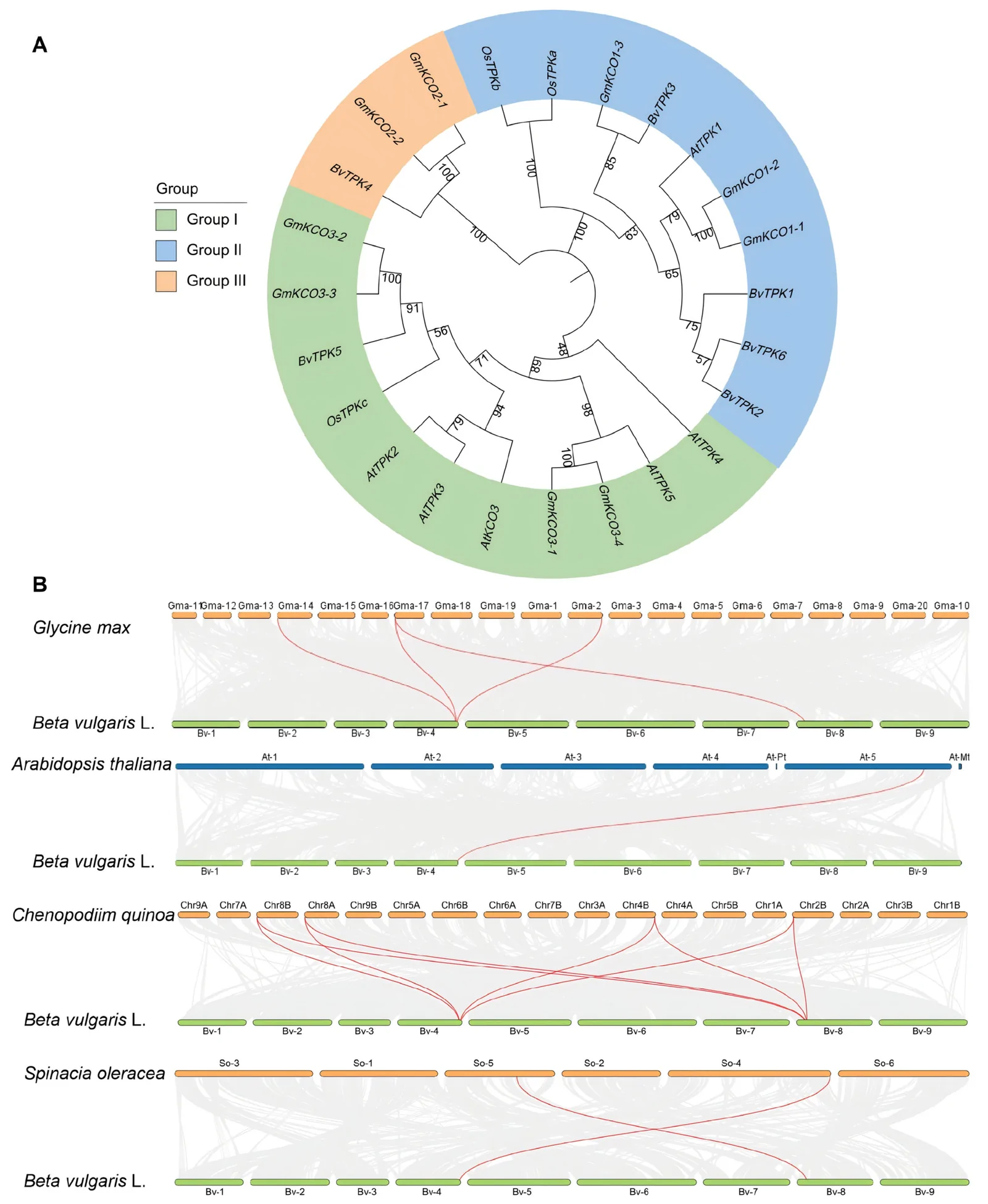
Phylogenetic and synteny analyses of TPK/KCO family members in sugar beet and representative plant species. (**A**) Neighbor-joining (NJ) phylogenetic tree of TPK/KCO family proteins from *Beta vulgaris*, *Arabidopsis thaliana*, *Glycine max*, and *Oryza sativa*. Bootstrap values were calculated from 1000 replicates. Groups I–III are indicated by green, blue, and orange backgrounds, respectively. (**B**) Synteny analysis of TPK/KCO family genes between *B. vulgaris* and *G. max*, *A. thaliana*, *C. quinoa*, and *S. oleracea*. Gray lines indicate syntenic blocks between species, whereas red lines highlight syntenic gene pairs involving TPK/KCO family members.

To investigate the evolutionary relationships among TPK/KCO family members, a phylogenetic tree was constructed using six *Arabidopsis* TPK/KCO proteins, three OsTPKs from rice, nine GmKCOs from soybean, and six BvTPKs from sugar beet. The TPK/KCO proteins were classified into three major groups (Groups I–III; Figure 2A). Group I included BvTPK5, OsTPKc, AtTPK2, AtTPK3, AtTPK4, AtTPK5, AtKCO3, GmKCO3-1, GmKCO3-2, GmKCO3-3, and GmKCO3-4. Within this group, BvTPK5 was positioned close to GmKCO3-2 and GmKCO3-3. Group II contained BvTPK1, BvTPK2, BvTPK3, and BvTPK6, together with AtTPK1, OsTPKa, OsTPKb, GmKCO1-1, GmKCO1-2, and GmKCO1-3. OsTPKa and OsTPKb clustered closely within this group, while BvTPK3 was positioned near GmKCO1-3. Group III consisted of BvTPK4, GmKCO2-1, and GmKCO2-2, with BvTPK4 forming a closely related branch with the two soybean proteins. Overall, the six BvTPKs were distributed among all three phylogenetic groups, with four members assigned to Group II and one member each assigned to Groups I and III, indicating substantial evolutionary diversification among BvTPK family members.

Interspecific synteny analysis was initially performed between sugar beet and soybean or *Arabidopsis*, two species included in the phylogenetic analysis. To further investigate the evolutionary conservation of the TPK/KCO family in sugar beet, quinoa (*Chenopodium quinoa*) and spinach (*Spinacia oleracea*), two phylogenetically related species within the Amaranthaceae, were additionally included for comparison (Figure 2B). A total of 15 syntenic gene pairs involving TPK/KCO family members were identified between sugar beet and the four species. Among the six *BvTPK* members, *BvTPK1* showed the most extensive syntenic relationships, with syntenic counterparts detected in all four species. *BvTPK3* exhibited syntenic relationships with homologous genes in soybean, quinoa, and spinach, whereas *BvTPK4* and *BvTPK5* showed syntenic relationships only with soybean. In contrast, no syntenic relationships were detected for *BvTPK2* or *BvTPK6*. Specifically, four syntenic gene pairs were identified between sugar beet and soybean, one between sugar beet and *Arabidopsis*, eight between sugar beet and quinoa, and two between sugar beet and spinach. Overall, the extent of syntenic conservation varied among individual *BvTPK* members, with *BvTPK1* and *BvTPK3* exhibiting broader syntenic relationships across species, suggesting that these two genes retain relatively conserved genomic contexts across the species examined.

### 2.3. Analysis of Conserved Motifs, Gene Structures, and Cis-Acting Regulatory Elements of the TPK/KCO Family in Sugar Beet

**Figure 3:**
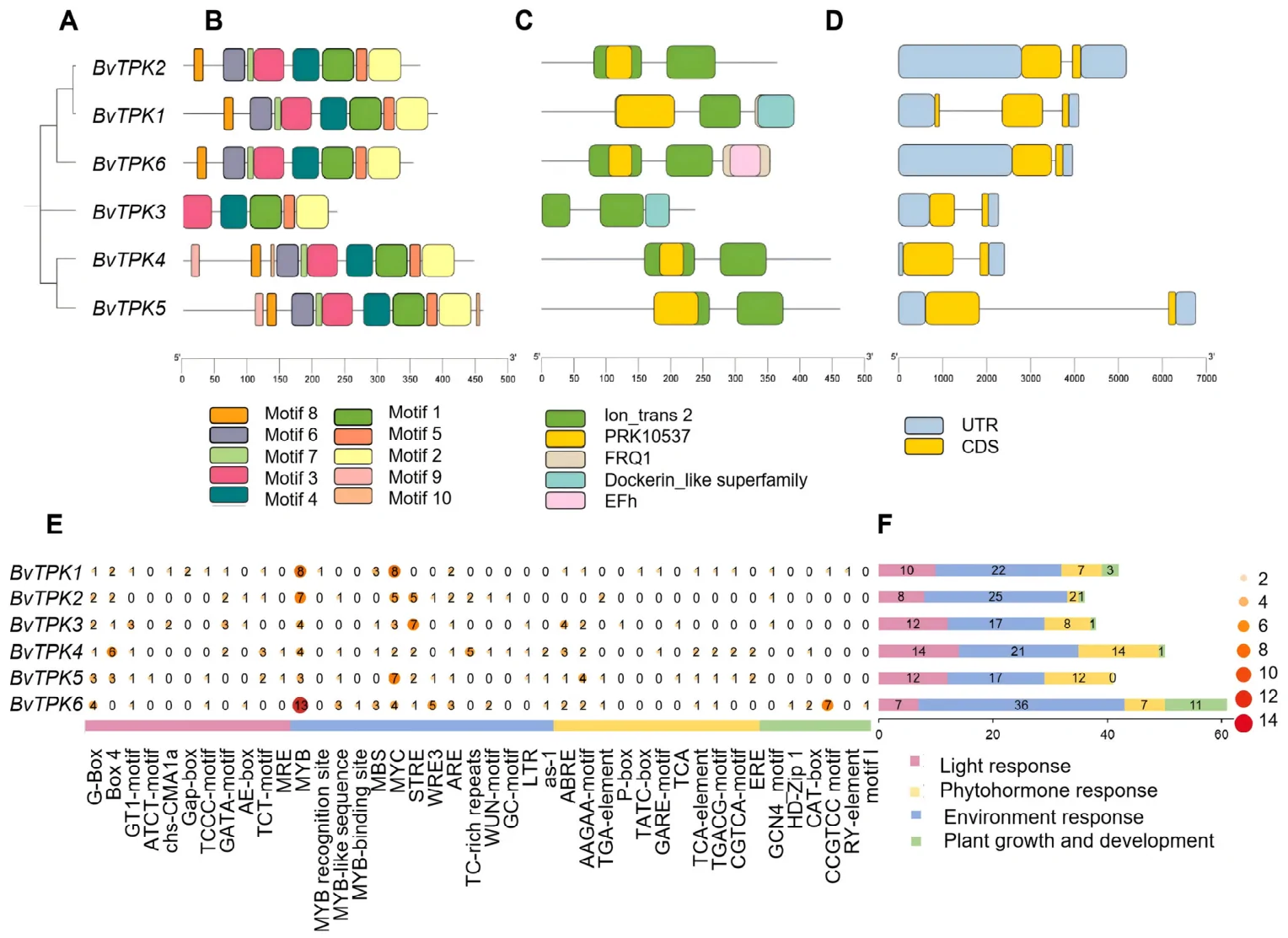
Phylogenetic relationships, protein structures, gene organization, and promoter cis-regulatory elements of the sugar beet TPK/KCO family. (**A**) A neighbor-joining phylogenetic tree of the six BvTPK proteins constructed with 1000 bootstrap replicates. (**B**) Distribution of conserved motifs in BvTPK proteins predicted using MEME. Colored boxes represent Motifs 1–10, and gray lines indicate the remaining protein sequences. (**C**) Conserved domain architecture of BvTPK proteins identified using the NCBI Conserved Domain Database. Colored boxes represent the annotated conserved domains, and gray lines indicate the remaining protein regions. (**D**) Exon–intron structures of the *BvTPK* genes. Light blue boxes, yellow boxes, and gray lines represent untranslated regions (UTRs), coding sequences (CDSs), and introns, respectively. (**E**) Distribution and abundance of cis-acting regulatory elements identified within the 2-kb regions upstream of the *BvTPK* genes. Numerical values and the sizes and colors of the circles indicate the copy number of each cis-element. (**F**) Functional classification and abundance of cis-acting regulatory elements in the *BvTPK* upstream regions. Pink, yellow, blue, and green represent light response, phytohormone response, environmental response, and plant growth and development, respectively.

Phylogenetic analysis classified the six BvTPK proteins into three distinct groups (Figure 3A). BvTPK1, BvTPK2, and BvTPK6 clustered within one group, BvTPK4 and BvTPK5 formed a second group, whereas BvTPK3 was placed in a distinct group, suggesting evolutionary divergence among TPK/KCO family members in sugar beet. Conserved motif analysis further revealed a combination of structural conservation and divergence among sugar beet TPK/KCO family members (Figure 3B). Members within the same phylogenetic group generally exhibited similar motif compositions and arrangements. In particular, BvTPK1, BvTPK2, and BvTPK6 displayed highly similar motif patterns, while BvTPK4 and BvTPK5 also shared comparable motif architectures. In contrast, BvTPK3 contained fewer motifs and exhibited a relatively distinct motif composition and arrangement among the family members. These results indicate that the TPK/KCO family in sugar beet retains overall structural conservation while exhibiting divergence among individual members. All BvTPK proteins contained the Ion_trans_2 conserved domain, whereas the PRK10537 domain was present in BvTPK1, BvTPK2, BvTPK4, BvTPK5, and BvTPK6 but absent from BvTPK3 (Figure 3C). Gene structures were also relatively conserved among all *BvTPK* genes, with two to three exons and one to two introns (Figure 3D).

Promoter analysis classified the identified cis-acting elements into four categories: light responsiveness, environmental stress responsiveness, phytohormone responsiveness, and plant growth and development. Environmental stress-related elements were the most abundant, accounting for approximately 41–59% of the elements in individual promoters (Figure 3E,F). MYB- and MYC-binding sites were detected in all six promoters. STREs occurred in all members except *BvTPK1*, AREs were present in all six genes, and LTRs were detected only in *BvTPK3*, *BvTPK4*, and *BvTPK5*. ABREs and AAGAA motifs were the most prevalent hormone-responsive elements and occurred in five members, followed by MeJA- and salicylic acid-responsive elements. Notably, *BvTPK6* contained seven CCGTCC motifs and showed the highest abundance of plant growth- and development-related elements, whereas the other genes contained only zero to three such elements. Overall, the promoter composition indicates that the TPK/KCO family in sugar beet is primarily associated with environmental stress and hormone responses.

### 2.4. Organ-Dependent and Salt-Responsive Expression Patterns of TPK/KCO Family Members in Sugar Beet

**Figure 4:**
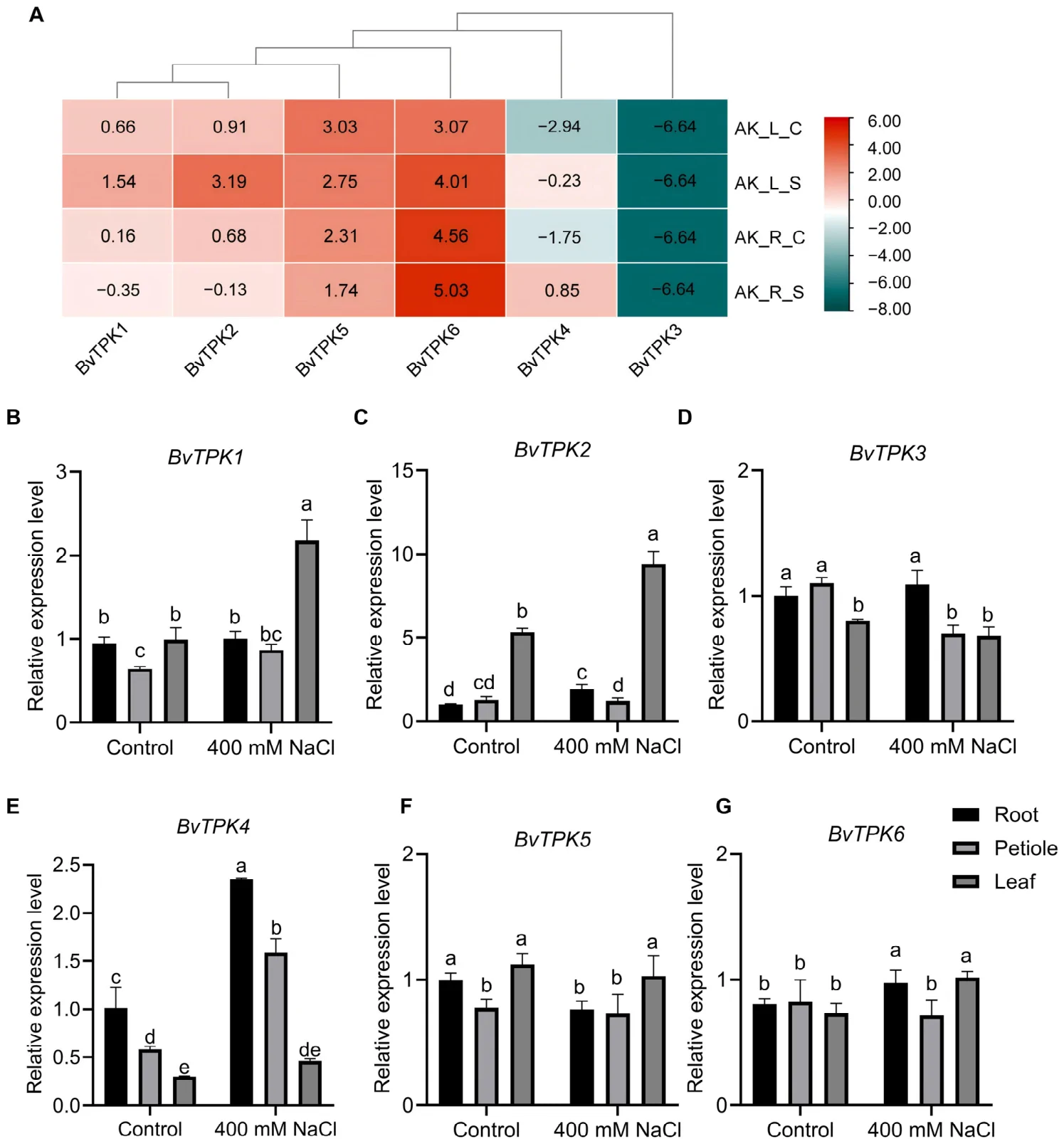
Organ-dependent and salt-responsive expression profiles of the sugar beet *BvTPK* gene family. (**A**) Hierarchical clustering heatmap showing the RNA-seq-derived expression profiles of *BvTPK*1–*BvTPK6* in sugar beet roots and leaves under control and salt-stress conditions. Expression values of zero were replaced with 0.01 before log_2_ transformation. Colors and numerical values within the cells represent log-transformed transcript abundance; no row-wise standardization was applied. AK_L_C and AK_L_S denote leaf samples under control and salt-stress conditions, respectively, whereas AK_R_C and AK_R_S denote root samples under control and salt-stress conditions, respectively. (**B**–**G**) RT–qPCR analysis of *BvTPK1*–*BvTPK6* expression in roots, petioles, and leaves under control conditions or following 400 mM NaCl treatment. Panels (**B**–**G**) correspond to *BvTPK1*–*BvTPK6*, respectively. Relative transcript abundance was calculated using the 2^−ΔΔCt^ method. Data are presented as the mean ± SD of three independent biological replicates (*n* = 3). Different lowercase letters indicate significant differences among organ–treatment combinations for each gene according to one-way analysis of variance followed by Duncan’s multiple range test (*p* < 0.05).

Transcriptomic data from the leaves and roots of the sugar beet cultivar ‘AK3018’ were analyzed to characterize the expression patterns of the *TPK/KCO family in sugar beet* under salt stress (Figure 4A). Under control conditions, the six *BvTPK* genes exhibited distinct organ-dependent expression profiles. *BvTPK5* and *BvTPK6* showed relatively high transcript abundance, whereas *BvTPK3* exhibited very low expression levels. *BvTPK1* and *BvTPK2* showed moderate expression, while *BvTPK4* exhibited relatively low expression in both leaves and roots. Following treatment with 400 mM NaCl, individual TPK/KCO family members exhibited distinct transcriptional responses. *BvTPK1* and *BvTPK2* were induced in leaves, whereas their expression levels decreased in roots. In contrast, *BvTPK4* was markedly induced in both leaves and roots. *BvTPK5* showed a slight decrease in both organs, *BvTPK6* exhibited increased expression in leaves and roots, and *BvTPK3* remained at a very low expression level under both control and salt-stress conditions.

RT-qPCR analysis was subsequently performed in roots, petioles, and leaves to further examine the salt-responsive expression patterns of the *BvTPK* genes (Figure 4B–G). Overall, the RT-qPCR results were partially consistent with the RNA-seq data, although several gene- and organ-dependent differences were observed. *BvTPK1* and *BvTPK2* were strongly induced in leaves under salt stress, in agreement with the RNA-seq results. However, *BvTPK2* expression decreased in roots according to RNA-seq, whereas RT–qPCR showed a slight increase. *BvTPK3* exhibited little change in roots but decreased expression in petioles and leaves under salt stress; although its transcript abundance was very low in the RNA-seq dataset, low but detectable expression was observed by RT–qPCR. *BvTPK4* was markedly induced in roots and petioles and showed a slight increase in leaves, generally consistent with its salt-induced expression pattern detected by RNA-seq. *BvTPK5* exhibited relatively minor changes, whereas *BvTPK6* showed modest increases in roots and leaves but decreased expression in petioles. Because petiole samples were not included in the RNA-seq dataset, expression changes in petioles could not be directly compared between the two methods. Importantly, both analyses consistently showed strong salt-induced expression of *BvTPK2* in leaves, with its relative expression level increasing from approximately 5.2 under control conditions to 9.3 after 400 mM NaCl treatment. Thus, despite some gene- and organ-dependent discrepancies, both datasets consistently supported the strong salt responsiveness of *BvTPK2* in leaves, providing the basis for its selection for subsequent functional analysis.

**Figure 5:**
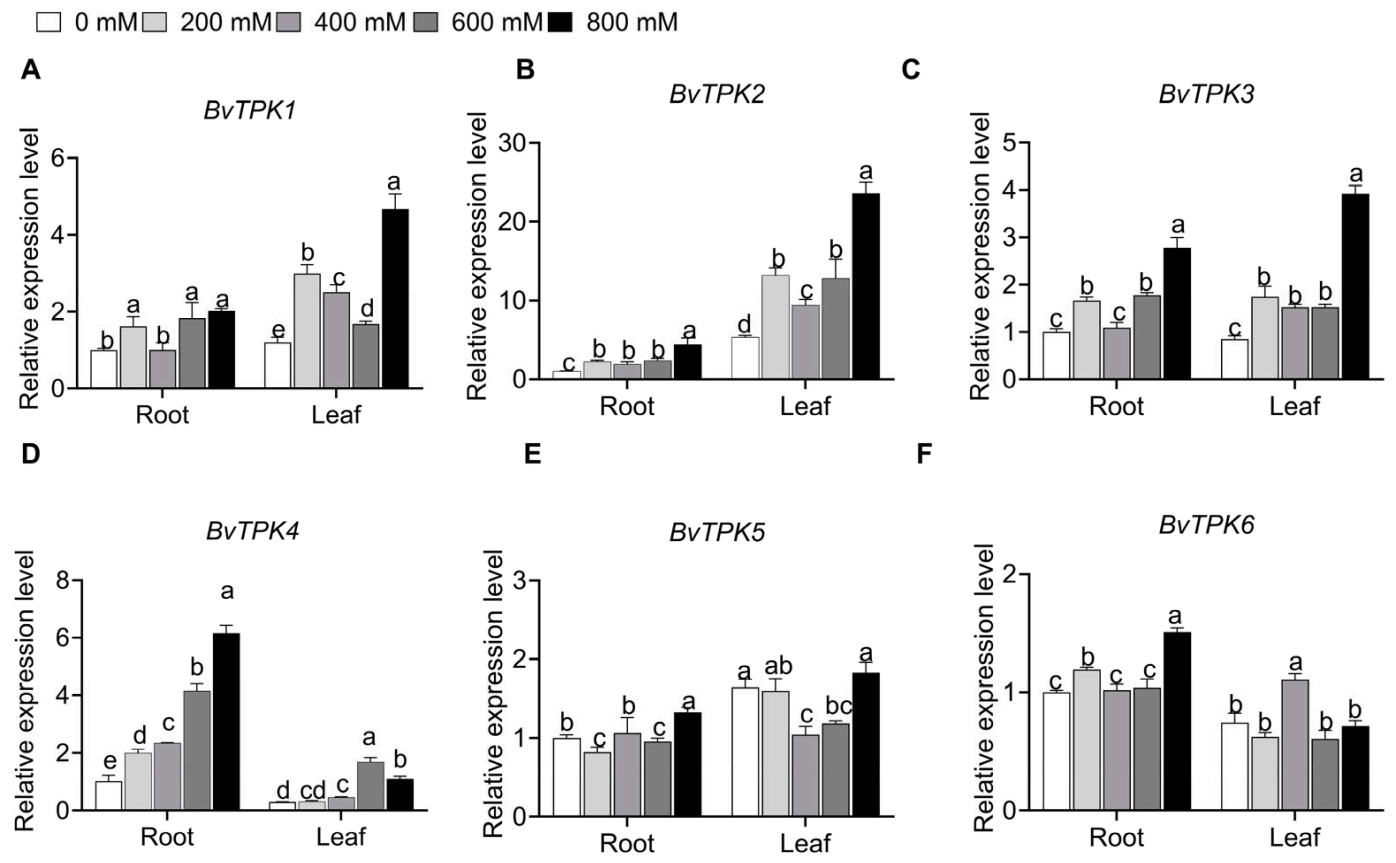
Expression profiles of *BvTPK* genes in sugar beet roots and leaves under different NaCl concentrations. Relative expression levels of *BvTPK1*–*BvTPK6* were determined in roots and leaves under 0, 200, 400, 600, and 800 mM NaCl treatments. (**A**–**F**) Expression patterns of *BvTPK1*, *BvTPK2*, *BvTPK3*, *BvTPK4*, *BvTPK5*, and *BvTPK6*, respectively. Data are presented as means ± SD of three biological replicates (*n* = 3). Different lowercase letters indicate significant differences among NaCl treatments within the same organ (one-way ANOVA followed by Duncan’s multiple range test, *p* < 0.05).

To further characterize the salt-responsive expression patterns of *BvTPK* genes under different NaCl concentrations, the relative expression levels of individual *BvTPK* genes were examined in sugar beet roots and leaves under different NaCl treatments. Among the six genes, *BvTPK1*, *BvTPK2*, *BvTPK3*, and *BvTPK4* exhibited varying degrees of concentration-responsive expression, whereas *BvTPK5* and *BvTPK6* showed relatively modest or organ-dependent changes in roots and leaves. Among these genes, *BvTPK2* showed a pronounced response to salt stress, with its expression generally increasing in both roots and leaves as the NaCl concentration increased. The response was particularly pronounced in leaves, where *BvTPK2* expression increased under 200–800 mM NaCl and peaked at 800 mM NaCl, reaching a relative expression level of approximately 24, about fourfold higher than that under control conditions. The response of *BvTPK1* was more evident in leaves, where its expression exhibited a fluctuating but overall increasing pattern and reached its highest level at 800 mM NaCl, whereas changes in roots were relatively limited. *BvTPK3* showed moderate fluctuations at lower NaCl concentrations but was markedly induced in both roots and leaves at 800 mM NaCl. Notably, *BvTPK4* expression showed a clear concentration-dependent increase in roots and reached its highest level at 800 mM NaCl. In leaves, *BvTPK4* expression increased with increasing NaCl concentration up to 600 mM, followed by a slight decrease at 800 mM. *BvTPK5* exhibited relatively modest fluctuations in both organs, whereas *BvTPK6* showed organ-dependent responses, with increased expression in roots at 800 mM NaCl and a relatively higher expression level in leaves at 400 mM NaCl. Overall, the *BvTPK* family exhibited distinct organ- and concentration-dependent responses to salt stress, with *BvTPK2* showing the strongest induction in leaves and *BvTPK4* displaying a pronounced concentration-dependent response in roots (Figure 5).

Collectively, the transcriptomic and RT–qPCR analyses revealed distinct organ and concentration-dependent expression patterns of *BvTPK* family members in response to salt stress. Although several family members responded to NaCl treatment, *BvTPK2* exhibited the most pronounced and consistent induction. Its expression was markedly induced in both roots and leaves across the tested NaCl concentrations, with a substantially stronger response in leaves, where transcript levels peaked at 800 mM NaCl. This expression profile was consistent with the transcriptomic data and further distinguished *BvTPK2* from the other family members. These findings identify *BvTPK2* as a prominent salt-responsive member of the *BvTPK* family and provide a clear basis for its selection for subsequent functional characterization.

### 2.5. BvTPK2 Is Localized to the Tonoplast

**Figure 6:**
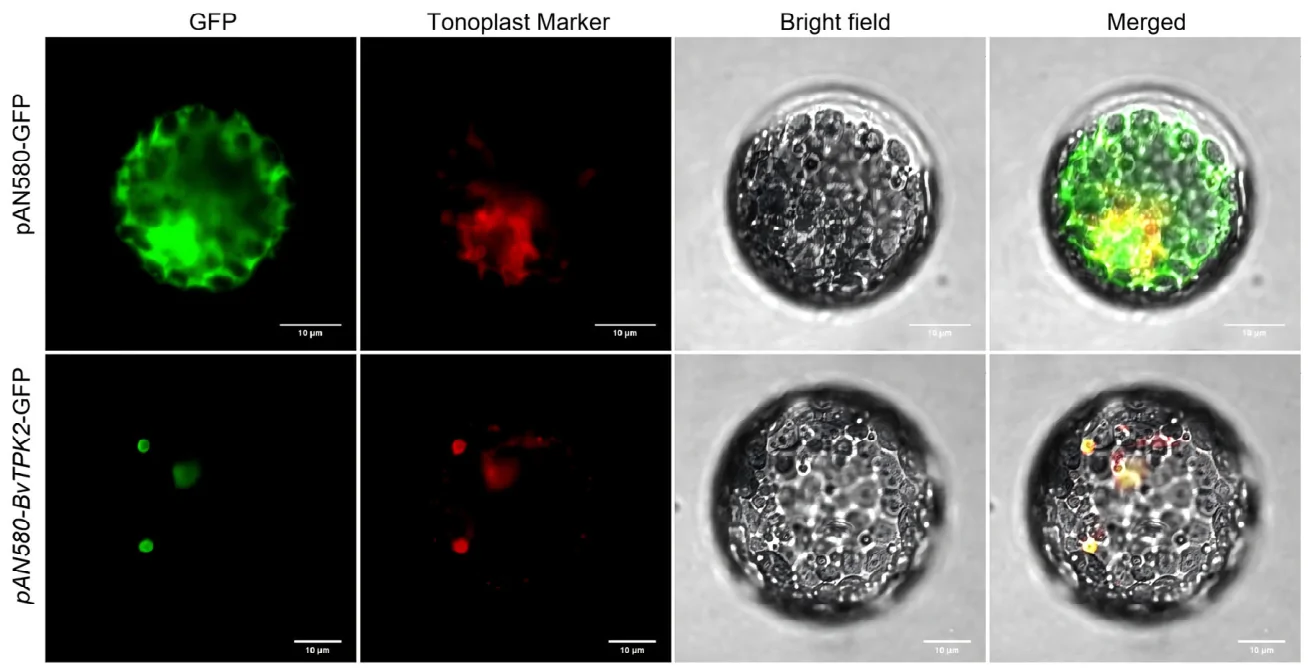
Subcellular localization of BvTPK2. Transient expression of pAN580-GFP (empty vector control) and pAN580-BvTPK2-GFP (green) in tobacco protoplasts, co-expressed with a tonoplast marker (red). The green fluorescence of pAN580-BvTPK2-GFP co-localized with the tonoplast marker signal, indicating tonoplast localization. Scale bar: 10 μm.

To determine the subcellular localization of BvTPK2, pAN580-BvTPK2-GFP was transiently expressed in tobacco protoplasts, with pAN580-GFP as the control and a tonoplast marker for colocalization analysis. Free GFP was diffusely distributed throughout the protoplast, whereas BvTPK2-GFP fluorescence was predominantly localized to the tonoplast and overlapped with the marker signal, producing a yellow signal in the merged image (Figure 6). These results demonstrate that BvTPK2 localizes to the tonoplast. The observed subcellular localization of BvTPK2 was consistent with its previously predicted localization.

### 2.6. Identification of BvTPK2-Overexpressing Arabidopsis Lines

**Figure 7:**
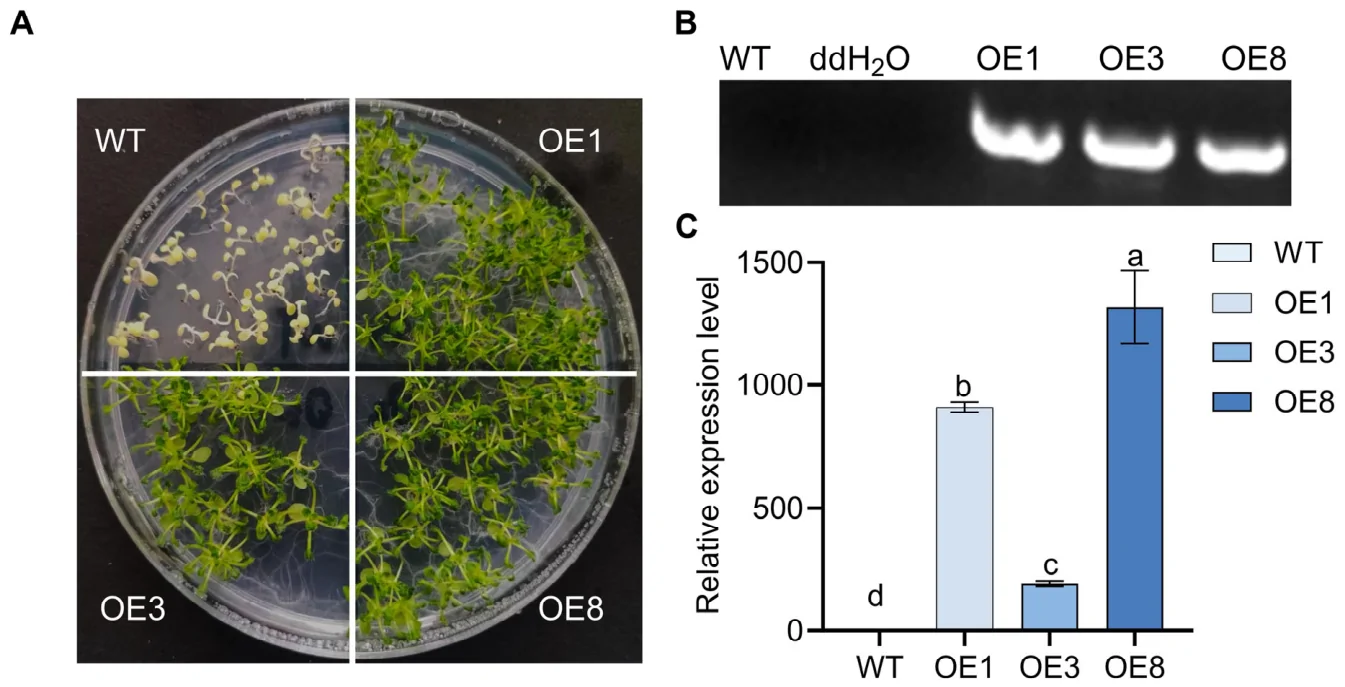
Identification of *BvTPK2*-overexpressing *Arabidopsis* lines. (**A**) Phenotypes of wild-type (WT) and three independent *BvTPK2*-overexpressing lines (OE1, OE3, and OE8) selected on medium containing 50 mg L^−1^ kanamycin. (**B**) Genomic PCR analysis confirming the presence of *BvTPK2* in the transgenic lines. WT and ddH_2_O were used as negative controls. (**C**) Relative expression levels of *BvTPK2* in WT and transgenic lines determined by RT–qPCR using the 2^−ΔΔCt^ method, with WT used as the calibrator (WT = 1). Data are presented as means ± SD (*n* = 3). Different lowercase letters indicate significant differences among genotypes (*p* < 0.05).

The pBI101-*BvTPK2* overexpression construct was generated and introduced into WT using the Agrobacterium-mediated floral dip method. Three independent kanamycin-resistant lines, OE1, OE3, and OE8, were selected and propagated to the T3 generation on medium containing 50 mg L^−1^ kanamycin (Figure 7A). Genomic PCR confirmed the presence of the *BvTPK2* transgene in all three lines (Figure 7B). RT–qPCR analysis further showed that *BvTPK2* transcript levels were significantly increased in the three transgenic lines compared with WT, although the expression levels varied among the lines (Figure 7C). These results verified the successful generation of independent *BvTPK2*-overexpressing *Arabidopsis* lines.

### 2.7. Effects of BvTPK2 Overexpression on Seed Germination and Early Seedling Survival Under Salt Stress

**Figure 8:**
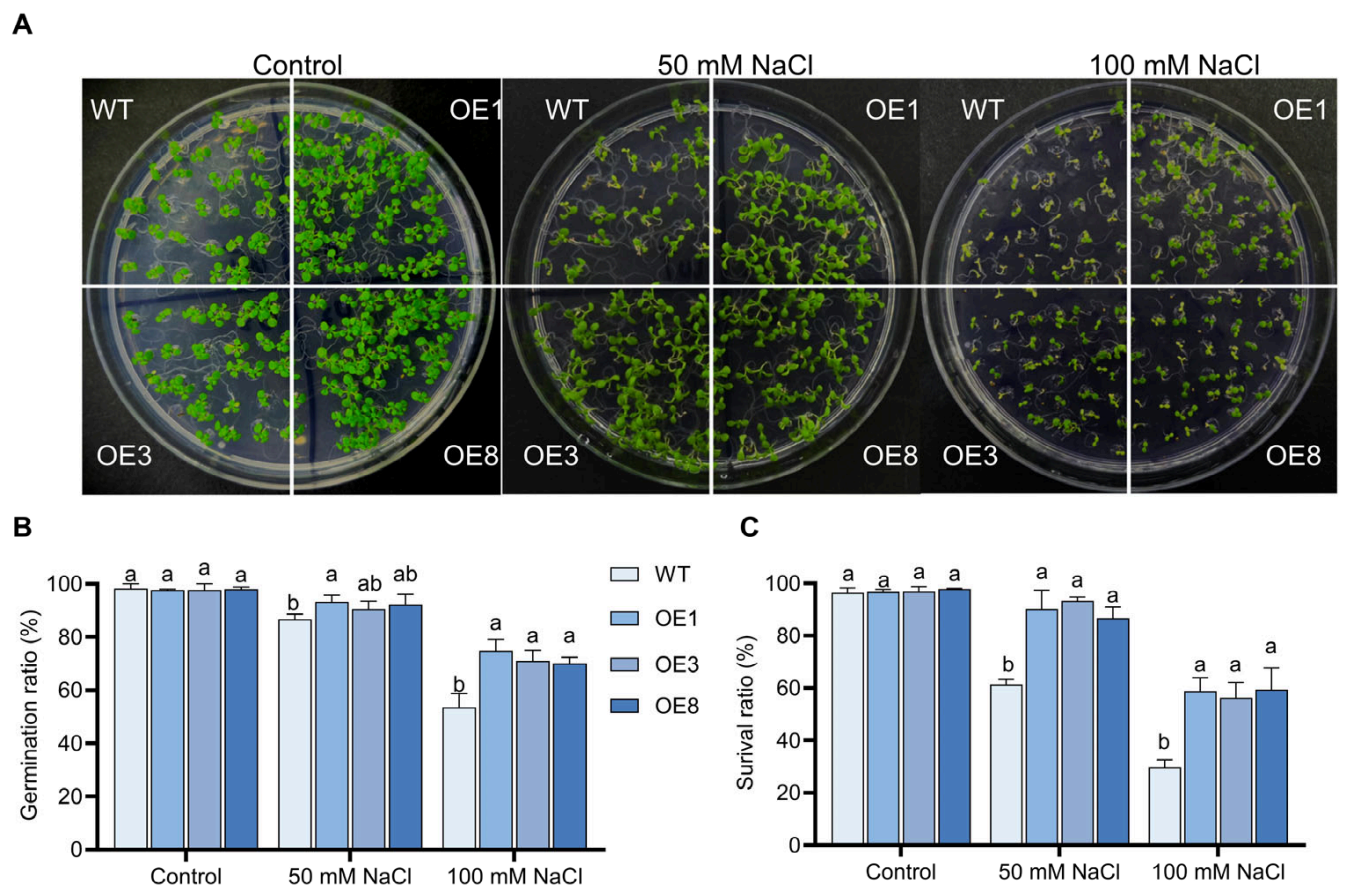
*BvTPK2* overexpression enhances salt tolerance in *Arabidopsis* during seed germination and early seedling growth. (**A**) Phenotypes of wild-type (WT) and three independent *BvTPK2*-overexpressing lines (OE1, OE3, and OE8) under control conditions and 50 or 100 mM NaCl treatment. (**B**) Germination rates of WT and *BvTPK2*-overexpressing lines under the indicated NaCl concentrations. (**C**) Survival rates of WT and *BvTPK2*-overexpressing lines under the indicated NaCl concentrations. Data are presented as means ± SD (*n* = 3). Different lowercase letters indicate significant differences among genotypes within the same treatment (*p* < 0.05).

To evaluate the role of *BvTPK2* in salt tolerance, seed germination and seedling survival of WT and *BvTPK2*-overexpressing *Arabidopsis* plants were evaluated under salt stress. Under control conditions, no significant differences in germination, survival, or growth were observed among the genotypes. Following treatment with 100 mM NaCl, seed germination and seedling survival were markedly reduced in all plants; however, the overexpression lines maintained significantly higher germination and survival rates than WT plants (Figure 8A–C). Collectively, these results indicate that *BvTPK2* overexpression enhances seed germination and seedling survival under salt stress.

**Figure 9:**
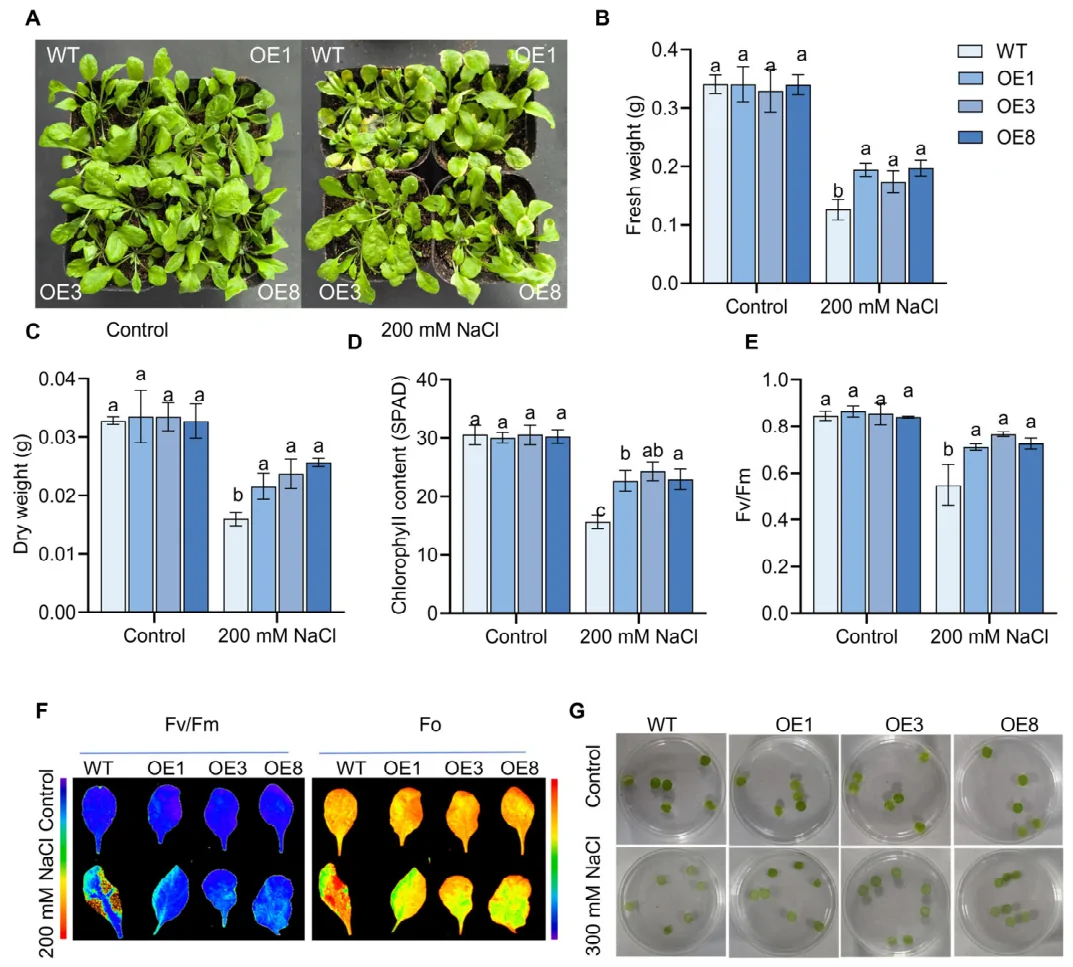
Effects of *BvTPK2* overexpression on growth and photosynthetic performance of *Arabidopsis* under salt stress. (**A**) Growth phenotypes of wild-type (WT) and three independent *BvTPK2*-overexpressing lines (OE1, OE3, and OE8) under control and 200 mM NaCl conditions. (**B**,**C**) Fresh weight (**B**) and dry weight (**C**) of WT and overexpression lines. (**D**) Relative chlorophyll content determined using a SPAD meter. (**E**) Maximum quantum efficiency of photosystem II (Fv/Fm). (**F**) Representative chlorophyll fluorescence images showing Fv/Fm and minimum fluorescence (Fo) under control and 200 mM NaCl conditions. (**G**) Detached-leaf disc senescence phenotypes of WT and *BvTPK2*-overexpressing lines under control conditions and after treatment with 300 mM NaCl for 5 days. Data are presented as the means ± SD (*n* = 3). Different lowercase letters indicate significant differences among genotypes within the same treatment (*p* < 0.05).

To evaluate the effects of salt stress on plant growth and photosynthetic performance, WT and *BvTPK2*-overexpressing *Arabidopsis* plants were evaluated under control and 200 mM NaCl conditions. Under control conditions, no significant differences in growth were observed between WT and *BvTPK2*-overexpressing lines. Under NaCl treatment, both WT and *BvTPK2*-overexpressing plants exhibited growth inhibition, leaf chlorosis, and wilting; however, these symptoms were more pronounced in WT plants (Figure 9A). Consistent with these phenotypic differences, WT plants had significantly lower fresh weight (Figure 9B) and dry weight (Figure 9C) than the *BvTPK2*-overexpressing lines under salt stress. Following treatment with 200 mM NaCl, *BvTPK2*-overexpressing plants exhibited significantly higher SPAD values than WT plants (Figure 9D), along with higher Fv/Fm values (Figure 9E) and stronger chlorophyll fluorescence signals (Figure 9F). Moreover, leaf discs from the *BvTPK2*-overexpressing lines exhibited less pronounced senescence than those from WT plants under salt stress (Figure 9G). Collectively, these results indicate that *BvTPK2* overexpression is associated with reduced growth inhibition and better maintenance of photosynthetic performance under salt stress, supporting a positive role for *BvTPK2* in the salt-stress response of *Arabidopsis*.

**Figure 10:**
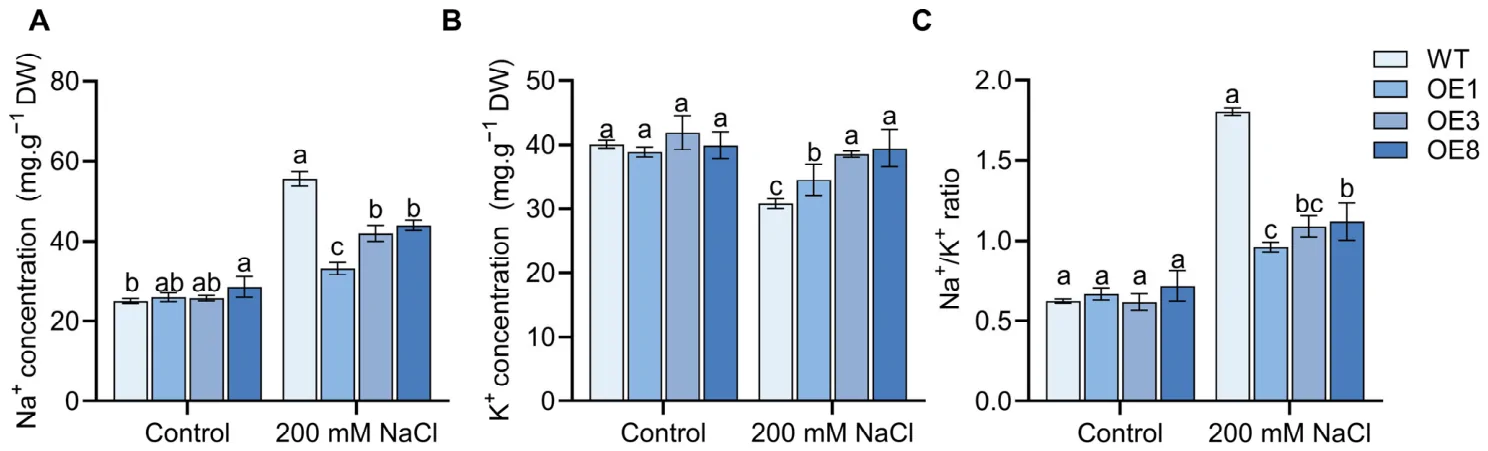
Effects of *BvTPK2* overexpression on Na^+^ and K^+^ accumulation in *Arabidopsis* under salt stress. (**A**) Na^+^ concentration, (**B**) K^+^ concentration, and (**C**) Na^+^/K^+^ ratio in WT and *BvTPK2*-overexpressing lines (OE1, OE3, and OE8) under control conditions and 200 mM NaCl treatment. Values are presented as means ± SD. (*n* = 3) Different lowercase letters indicate significant differences among genotypes within the same treatment according to one-way ANOVA followed by Duncan’s multiple range test (*p* < 0.05).

Under control conditions, Na^+^ and K^+^ concentrations and Na^+^/K^+^ ratios did not differ significantly between WT and the *BvTPK2*-overexpressing lines, although minor differences in Na^+^ concentration were observed (Figure 10A–C). Under 200 mM NaCl treatment, the overexpressing lines accumulated significantly less Na^+^ and maintained relatively higher K^+^ concentrations than WT (Figure 10A,B). Consequently, their Na^+^/K^+^ ratios were significantly lower than the WT ratio (Figure 10C). These results indicate that *BvTPK2* overexpression restricts Na^+^ accumulation and enhances K^+^ retention under salt stress, thereby contributing to the maintenance of Na^+^/K^+^ homeostasis.

## 3. Discussion

The six BvTPK proteins identified in this study exhibited considerable variation in their physicochemical properties, suggesting that members of this family may have diverged in protein composition, stability, and adaptation to membrane environments, potentially contributing to their functional diversification. The TPK/KCO family is relatively conserved in size across plant species, with 6, 3, 9, 7, and 8 members identified in *Arabidopsis thaliana*, *Oryza sativa*, *Glycine max*, *Gossypium hirsutum*, and *Brassica rapa*, respectively [8,12,14,19,20]. In this study, six *BvTPK* genes were identified in sugar beet, indicating no substantial family expansion of the TPK/KCO family. Phylogenetic analysis divided the TPK/KCO proteins from *Arabidopsis*, rice, soybean, and sugar beet into three major clades. Four BvTPK members, BvTPK1, BvTPK2, BvTPK3, and BvTPK6, were assigned to Group II together with AtTPK1, OsTPKa, OsTPKb, and soybean KCO1 members. In contrast, BvTPK5 was placed in Group I with OsTPKc, several *Arabidopsis* TPK/KCO proteins, and soybean KCO3 members, whereas BvTPK4 clustered within Group III together with soybean KCO2 members. The distribution of BvTPK proteins across all three clades suggests substantial diversification within the sugar beet TPK/KCO family, while their clustering with homologous proteins from other species also indicates a degree of evolutionary conservation among plant TPK/KCO members. Synteny analysis further revealed marked differences in the conservation of individual *BvTPK* members across species. *BvTPK1* retained syntenic relationships with homologous genes in all four species examined, whereas *BvTPK3* showed syntenic relationships with soybean, quinoa, and spinach. In contrast, *BvTPK4* and *BvTPK5* exhibited more restricted syntenic relationships, and no detectable synteny was observed for *BvTPK2* or *BvTPK6*. Notably, *BvTPK1* and *BvTPK2* are both located on chromosome 4 but displayed markedly different syntenic patterns, indicating that the conservation of individual *BvTPK* genes is not necessarily associated with chromosomal location. These differences may reflect lineage-specific gene retention or loss, as well as genomic rearrangements during species divergence. Together with the phylogenetic analysis, the synteny results suggest that the sugar beet TPK/KCO family has retained conserved features while undergoing member-specific diversification.

Canonical TPK/KCO family proteins generally contain four transmembrane helices, two pore-forming domains, and Ca^2+^-binding EF-hand motifs, whereas KCO3-like members usually contain only two transmembrane helices and one pore domain [7,21,22]. All BvTPK proteins contained the conserved Ion_trans_2 domain, whereas PRK10537 was detected in BvTPK1, BvTPK2, BvTPK4, BvTPK5, and BvTPK6 but was absent from BvTPK3. Their promoters were enriched in light-, phytohormone-, and abiotic stress-responsive cis-elements, including elements associated with abscisic acid, jasmonic acid, gibberellin, auxin, salicylic acid, drought, low temperature, and defense responses. Similar cis-element profiles have been reported for other K^+^ transport-related genes and the KCO family in *B. rapa* [20,23]. These findings suggest that TPK/KCO family members may integrate environmental, hormonal, and developmental signals to regulate ion homeostasis, whereas variation in promoter composition may underlie their distinct organ-dependent and stress-responsive expression patterns.

Plant TPK/KCO family members exhibit organ-specific expression patterns and differential responses to abiotic stress. For example, AtTPK4 (KCO4) is localized to the plasma membrane and is predominantly expressed in pollen [24]. Soybean *KCO1* and *KCO3* are differentially expressed during seed development, whereas strawberry *FaTPK1* is mainly expressed in fruit and contributes to fruit ripening and soluble compound accumulation. Tobacco *NtTPKa* is induced by K^+^ deficiency, while *BraKCO* genes in Chinese cabbage show distinct expression patterns across roots, stems, leaves, flowers, and siliques [14,20,25,26,27]. Similarly, the six *BvTPK* genes exhibited distinct expression patterns and salt responsiveness among sugar beet roots, petioles, and leaves. Notably, *BvTPK2* was markedly induced by salt stress in roots and leaves, suggesting that it is a key candidate involved in the salt stress response.

Subcellular localization is a key determinant of TPK channel function. *Arabidopsis* AtTPK1 and tobacco NtTPK1 are localized to the tonoplast; *NtTPK1* mediates highly selective K^+^ transport regulated by cytosolic Ca^2+^ and pH and is induced by salt and hyperosmotic stress. In rice, OsTPKa and OsTPKb target lytic and protein storage vacuoles, respectively, indicating localization-dependent functional specialization [12,21,26,28]. BvTPK2 was likewise localized to the tonoplast, providing spatial support for its potential involvement in vacuolar ion homeostasis.

Previous studies have demonstrated that TPK-type K^+^ channels play important roles in plant responses to salt and other abiotic stresses. In *Arabidopsis*, salt stress induces phosphorylation of AtTPK1, whereas *tpk1* mutants exhibit increased salt sensitivity [11]. In rice, reduced expression of *OsTPKa* impairs seed germination and accelerates leaf wilting, chlorosis, and chlorophyll loss under salt stress [13], Conversely, overexpression of *OsTPKa* improves plant growth under salt and drought stress and is associated with reduced Na^+^ accumulation and improved K^+^ homeostasis, whereas overexpression of *OsTPKb* enhances tolerance to K^+^ deficiency and osmotic or drought stress by promoting K^+^ uptake and maintaining cellular K^+^ balance [18,29,30]. In addition, heterologous expression of poplar *PeTPK1* in tobacco BY-2 cells improves growth under salt stress and is associated with reduced Na^+^ accumulation and enhanced K^+^ retention [17]. Strawberry *FaTPK1* also regulates vacuolar solute accumulation and plant growth, supporting a role for TPK channels in ionic and osmotic homeostasis [25]. Collectively, these findings indicate that TPK channels contribute to stress adaptation by supporting plant growth and maintaining cellular K^+^ and Na^+^ homeostasis. In the present study, *BvTPK2* overexpression enhanced seed germination and seedling survival, alleviated growth inhibition, and increased fresh and dry biomass in *Arabidopsis* under salt stress. The overexpressing lines also accumulated less Na^+^, maintained relatively higher K^+^ concentrations, and exhibited lower Na^+^/K^+^ ratios than WT, indicating a greater capacity to maintain ionic homeostasis under salt stress. These changes are consistent with the functions reported for *OsTPKa*, *OsTPKb*, and *PeTPK1*, suggesting that reduced Na^+^ accumulation and improved K^+^ retention may contribute to TPK-mediated stress tolerance. In addition, the *BvTPK2*-overexpressing lines maintained higher chlorophyll content and Fv/Fm values under salt stress, indicating better preservation of photosynthetic function. Since excessive Na^+^ accumulation and K^+^ loss can disrupt cellular metabolism and photosynthetic processes, the improved ionic balance in the overexpressing lines may contribute to their enhanced growth and photosynthetic performance. It should be noted that the high NaCl concentrations used for stress induction may simultaneously trigger osmotic and ionic stress. Although the present study mainly focused on ion homeostasis and salt-responsive regulation, whether *BvTPK2* contributes to osmotic adjustment and water homeostasis remains an interesting question for future investigation.

Given its tonoplast localization, BvTPK2 may be involved in vacuolar K^+^ homeostasis and cellular ion balance under salt stress. However, the current ion content data do not directly demonstrate its ion transport activity or transport direction. Further ion flux and electrophysiological analyses are needed to determine the ion transport properties of BvTPK2 and clarify their relationship with salt tolerance.

## 4. Materials and Methods

### 4.1. Plant Materials and Treatments

Seeds of the sugar beet (*Beta vulgaris* L.) cultivar ‘AK3018’ were purchased from Beijing Jinyangpu Agricultural Co., Ltd. (Beijing, China). No wild or protected plant materials were collected, and all experimental procedures complied with relevant institutional, national, and international guidelines. Wild-type *Arabidopsis thaliana* Columbia-0 (Col-0) seeds were maintained in our laboratory.

Sugar beet seeds were sown in plug trays and grown in a controlled-environment chamber (custom-built by Shandong Keyi Experimental Equipment Co., Ltd., Jinan, China) under a 16-h light/8-h dark photoperiod at 26/22 °C (day/night temperature) with a light intensity of 10,000 lux. At the four- to six-leaf stage, uniformly developed seedlings were treated with 0, 200, 400, 600, and 800 mM NaCl solutions by root irrigation. Each pot was irrigated once with 75 mL of the corresponding NaCl solution, and irrigation was continued until a small amount of solution drained from the bottom of the pot to ensure relatively uniform distribution of salt in the substrate. No additional NaCl solution was applied during the 7-day treatment period. Roots, petioles, and leaves were collected, immediately frozen in liquid nitrogen, and stored at −80 °C. Wild-type *Arabidopsis thaliana* Columbia-0 (Col-0) seeds were surface-sterilized and germinated on Murashige and Skoog (MS) medium. Fifteen-day-old seedlings were transplanted into plug trays and maintained under the same controlled conditions until use.

### 4.2. Identification and Characterization of the Sugar Beet TPK/KCO Family

The sugar beet genome, coding sequences (CDSs), protein sequences, and GFF3 annotation file were downloaded from the Sugar Beet Genome Database (http://www.ebi.ac.uk/ena/data/view/GCA_000511025.2 (accessed on 18 August 2026)). The hidden Markov model profile of the TPK/KCO family ion-channel domain (PF07885) was obtained from Pfam (http://pfam.xfam.org/) and used to search the sugar beet protein database with HMMER v3.0 (http://hmmer.org/) at an E-value threshold of 1 × 10^−5^. Redundant sequences were removed manually, and the remaining candidates were subjected to a second-round BLASTP search. Conserved domains were verified using SMART (http://smart.embl-heidelberg.de/ (accessed on 18 August 2026)). Candidates containing characteristic TPK/KCO conserved domains and structural features were retained for further analysis. Multiple sequence alignment of BvTPK proteins was performed using DNAMAN software (version 6.0; Lynnon Biosoft, San Ramon, CA, USA) to analyze conserved amino acid residues and characteristic sequence motifs of the TPK/KCO family. Incomplete proteins and candidates lacking the characteristic TPK/KCO ion-channel domain were excluded. The amino acid length, molecular weight, theoretical isoelectric point, instability index, aliphatic index, and grand average of hydropathicity (GRAVY) of each BvTPK protein were calculated using TBtools-II (version 2.466). Chromosomal positions were extracted from the GFF3 annotation file and visualized with TBtools-II. Subcellular localization was predicted using WoLF PSORT (https://wolfpsort.hgc.jp/ (accessed on 19 August 2026)).

### 4.3. Phylogenetic and Synteny Analyses of the TPK/KCO Family

The amino acid sequences of TPK/KCO family proteins from sugar beet (*Beta vulgaris* L.), *Arabidopsis thaliana*, soybean (*Glycine max*), and rice (*Oryza sativa*) were aligned using MEGA version 7.0. A neighbor-joining (NJ) phylogenetic tree was constructed with 1000 bootstrap replicates using the default settings. Syntenic relationships of TPK/KCO family genes among sugar beet, soybean, *A. thaliana*, spinach (*Spinacia oleracea*), and quinoa (*Chenopodium quinoa*) were analyzed and visualized using TBtools-II with default parameters.

### 4.4. Conserved Motif, Gene Structure, and Promoter Cis-Regulatory Element Analyses

Conserved motifs in BvTPK proteins were identified using MEME Suite (http://meme-suite.org/tools/meme (accessed on 19 August 2026)), with the maximum number of motifs set to 10. Conserved domains were annotated using the NCBI Conserved Domain Search. Motif distribution, domain architecture, and exon–intron organization were visualized using TBtools-II. For promoter analysis, the 2-kb sequences upstream of the translation start codon of each *BvTPK* gene were extracted and analyzed using PlantCARE (http://bioinformatics.psb.ugent.be/webtools/plantcare/html/ (accessed on 19 August 2026)) to predict cis-regulatory elements. Their distribution and abundance were visualized using TBtools-II.

### 4.5. Expression Analysis of BvTPK Genes by RT–qPCR

FPKM values obtained from the sugar beet salt-stress transcriptome dataset [31] were log_2_-transformed after zero values were replaced with 0.01. The transformed data were visualized using GraphPad Prism (version 10.1.2; GraphPad Software, Boston, MA, USA) to characterize the expression patterns of *BvTPK* genes under salt stress. RT–qPCR was further performed to determine the relative expression levels of the six *BvTPK* genes. For organ expression analysis, roots, petioles, and leaves were collected from plants treated with 0 or 400 mM NaCl for 7 d. The 400 mM NaCl treatment was consistent with the salt-stress condition used for the previously reported transcriptomic analysis of the same sugar beet cultivar, AK3018, allowing the RT-qPCR results to be directly compared with the transcriptomic data. For salt concentration-response analysis, roots and leaves were collected after treatment with 0, 200, 400, 600, or 800 mM NaCl for 7 d, with the concentration range selected based on a previous salt-responsive expression study of *BvPPase* in sugar beet [32]. Based on preliminary salt gradient experiments, representative phenotypes of sugar beet plants exposed to different NaCl concentrations are provided in Supplementary Figure S1. Gene-specific primers were designed based on the corresponding gene sequences (Table S1), with *BvActin* used as the internal reference gene. Total RNA was extracted from each organ using TransZol Plant reagent according to the manufacturer’s instructions and reverse-transcribed into cDNA. The cDNA was diluted tenfold and used for RT–qPCR. RT-qPCR was performed using a CFX Connect Real-Time PCR Detection System (Bio-Rad Laboratories, Inc., Hercules, CA, USA). Three independent biological replicates were analyzed for each treatment, each with three technical replicates. Relative transcript levels were calculated using the 2^−ΔΔCt^ method.

### 4.6. Subcellular Localization of BvTPK2

The full-length coding sequence of *BvTPK2* was amplified by PCR using the gene-specific primers BvTPK2-GFP-F/R and inserted into the pAN580 vector linearized with *Bam*HI and *Xba*I to generate the pAN580-BvTPK2 construct. Mesophyll protoplasts were isolated from leaves of 15–20-day-old *Nicotiana benthamiana* plants. The leaves were incubated in an enzyme solution containing 1.5% (*w*/*v*) cellulase R10 and 0.4% (*w*/*v*) macerozyme R10 at 24 °C for 4 h. After digestion, the suspension was filtered through a 40 μm nylon mesh and centrifuged at 300 rpm for 3 min. The collected protoplasts were washed twice with pre-cooled W5 solution, gently resuspended in MMG solution, and adjusted to approximately 2 × 10^5^ cells mL^−1^ for subsequent PEG-mediated transformation. For transient expression, 10 μL of plasmid DNA solution was added to 100 μL of protoplast suspension, followed by an equal volume of PEG4000 solution. After transformation, the protoplasts were washed and resuspended in approximately 100 μL of W5 solution. The pAN580-BvTPK2 construct was co-transformed with an mKate-tagged AtTPK1 tonoplast marker, as AtTPK1 has been reported to localize to the vacuolar membrane [8]. Fluorescence signals were visualized by confocal laser-scanning microscopy (Nikon C2-ER; Nikon Corporation, Tokyo, Japan) using excitation wavelengths of 488 and 561 nm for GFP and the tonoplast marker, respectively.

### 4.7. Salt Tolerance Assays of Transgenic Arabidopsis

The *BvTPK2* open reading frame was inserted into the *Xba*I-linearized pBI101km-35S::GUS-Hm vector by homologous recombination. The verified construct was introduced into Agrobacterium tumefaciens strain GV3101 and subsequently transformed into wild-type (WT) *Arabidopsis thaliana* using the floral-dip method. T_1_ progeny were selected on MS medium containing 50 mg L^−1^ kanamycin and subsequently self-pollinated to obtain the T_3_ generation. Transgenic-positive T3 plants were identified by PCR using the Kan primer pair targeting the kanamycin-resistance marker. *BvTPK2* expression was quantified by RT–qPCR using AtActin as the reference gene and the 2^−ΔΔCt^ method. Three homozygous high-expression lines were selected for subsequent experiments. Seeds of homozygous transgenic *Arabidopsis thaliana* lines were surface-sterilized and stratified at 4 °C for 2 d before being sown on Murashige and Skoog (MS) medium supplemented with 0, 50, or 100 mM NaCl. For each genotype and treatment, approximately 50 seeds were sown for each biological replicate, and three independent biological replicates were performed. Germination was recorded after 7 d, and seeds were considered germinated when the radicle emerged. Survival rates were determined after 14 d based on the presence of green and normally growing seedlings. The 50 and 100 mM NaCl concentrations were selected based on preliminary experiments. For salt stress treatment at the adult stage, 20-day-old seedlings were transferred to soil and grown for another 20 d before treatment. Plants were irrigated once with either water or 200 mM NaCl solution, and phenotypic and physiological measurements were performed 7 d after this single treatment. Plants were irrigated once with either water or 200 mM NaCl solution, and phenotypic changes were recorded after salt treatment. The 200 mM NaCl concentration was selected based on a previous salt-tolerance study in transgenic *Arabidopsis* [33]. Plant phenotypes were photographed, and fresh and dry weights were determined. For biomass measurements, individual plants were measured separately, and three biological replicates were analyzed for each genotype and treatment. For the detached-leaf senescence assay, leaf discs (1 cm in diameter) were treated with 300 mM NaCl for 5 days to accelerate salt-induced senescence and enhance phenotypic differences among genotypes, following a previously reported protocol [33]. Senescence was assessed qualitatively based on salt-induced chlorosis and loss of green coloration. Leaf discs were photographed under identical conditions after treatment, and green color retention was used for comparison among genotypes. Relative chlorophyll content was measured using a SPAD-502 m, and the maximum quantum yield of photosystem II (Fv/Fm) was determined using a chlorophyll fluorescence imaging system. Plant samples were rinsed with deionized water, blotted dry, and oven-dried at 80 °C to constant weight. The dried samples were ground into powder, and a known amount was digested with 10% nitric acid. After cooling, the digest was diluted to a defined volume with deionized water. Na^+^ and K^+^ concentrations were determined using a flame photometer with standard calibration curves. Ion contents were calculated based on the dilution factors and sample dry weights. Three independent biological replicates were analyzed for each treatment. For ion-content determination, tissues from eight plants were pooled to constitute one biological replicate, and three independent biological replicates were analyzed for each genotype and treatment.

### 4.8. Statistical Analysis

Data are presented as means ± standard deviations (SDs) of three independent biological replicates unless otherwise stated. Statistical analyses were performed using IBM SPSS Statistics 26.0. Significant differences were assessed by one-way analysis of variance (ANOVA) followed by Duncan’s multiple range test at *p* < 0.05. Graphs were generated using GraphPad Prism and Microsoft Excel.

## 5. Conclusions

In summary, this study identified six members of the TPK/KCO family in *Beta vulgaris* L. with distinct structural features, regulatory elements, and expression patterns. The TPK/KCO family members in sugar beet exhibited organ-dependent and salt concentration-dependent expression patterns, with *BvTPK2* showing a pronounced salt-induced response, particularly in leaves. Subcellular localization analysis demonstrated that BvTPK2 localized to the tonoplast. Moreover, heterologous overexpression of *BvTPK2* significantly enhanced seed germination and seedling survival, improved plant growth and biomass, and maintained better photosynthetic performance in *Arabidopsis* under salt stress. The overexpressing lines also accumulated less Na^+^, maintained relatively higher K^+^ concentrations, and exhibited lower Na^+^/K^+^ ratios than WT. These results support the involvement of *BvTPK2* in the salt-stress response. These findings provide new insights into the characteristics and stress-responsive functions of the TPK/KCO family in sugar beet and identify *BvTPK2* as a candidate gene associated with salt tolerance for further functional investigation.

## Figures and Tables

**Table 1:** Physicochemical properties of members of the TPK/KCO family in sugar beet.

Gene ID	Gene Name	Protein Size (aa)	MW (kDa)	pI	Instability Index	Aliphatic Index	GRAVY	Subcellular Localization
*BVRB_4g090170*	*BvTPK1*	391	43.75	6.05	34.34	118.82	0.435	Plasma membrane
*BVRB_4g090180*	*BvTPK2*	364	40.46	5.63	41.33	105.74	0.235	Plasma membrane, vacuole
*BVRB_8g184500*	*BvTPK3*	237	26.65	5.52	27.07	109.75	0.392	Vacuole
*BVRB_9g203980*	*BvTPK4*	447	50.57	8.81	28.93	99.69	−0.126	Plasma membrane, endoplasmic reticulum
*BVRB_002500*	*BvTPK5*	461	51.48	8.07	47.91	101.43	0.015	Plasma membrane
*BVRB_003720*	*BvTPK6*	354	39.33	8.08	40.00	106.64	0.247	Plasma membrane

## Data Availability

Dataset available on request from the authors. The raw data supporting the conclusions of this article will be made available by the authors on request.

## Supplementary Materials

The following supporting information can be downloaded at https://www.mdpi.com/article/10.3390/plants15203156/s1, Figure S1: Phenotypic responses of sugar beet plants treated with different NaCl concentrations. Sugar beet plants were treated with different concentrations of NaCl (0, 200, 400, 600, and 800 mM) for 7 days. Representative photographs show the concentration-dependent effects of salt stress on plant growth and morphology. Figure S2: Multiple sequence alignment of BvTPK proteins. Multiple sequence alignment of six BvTPK proteins (BvTPK1-BvTPK6) was performed using DNAMAN. Conserved amino acid residues are highlighted according to sequence similarity. The conserved pore-forming region and the characteristic GYGD selectivity filter motif of TPK/KCO family proteins are indicated by red boxes. The alignment revealed conserved sequence features among BvTPK proteins, supporting their classification as members of the TPK/KCO family. Table S1: Primers used for qRT-PCR and PCR amplification. Table S2: Protein names, gene identifiers, accession numbers, and database sources of TPK/KCO proteins used for phylogenetic analysis. Supplementary File S1: Amino acid sequences of TPK/KCO family proteins from sugar beet, *Arabidopsis*, soybean, and rice used for phylogenetic tree construction.
