# Supplementary material for: Genome-Wide Identification of the Two-Pore Potassium Channel Gene Family in Sugar Beet (Beta vulgaris L.) and Functional Analysis of BvTPK2 in Salt Tolerance
Source: Plants (Basel). 2026 Oct 15;15(20):3156. doi: 10.3390/plants15203156 (PMC13709052; doi:10.3390/plants15203156)
Supplement: Supplementary file 1 [file plants-15-03156-s001.zip › Supplementary Figures S1-S2.pdf]

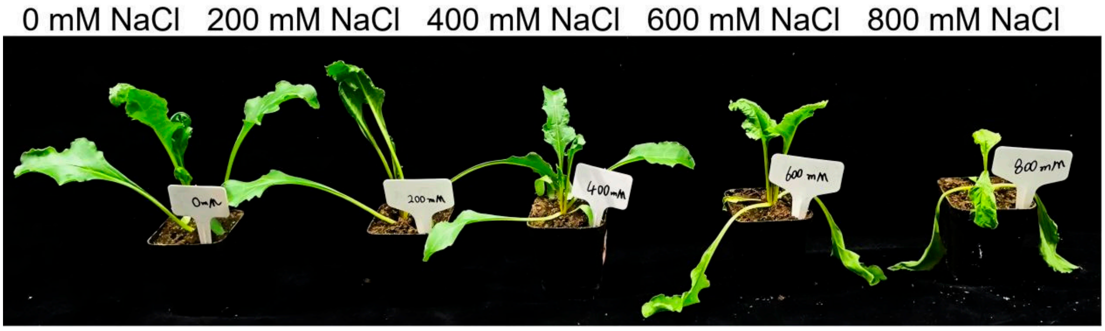

Figure S1. Phenotypic responses of sugar beet plants treated with different NaCl concentrations. Sugar beet plants were treated with different concentrations of NaCl (0, 200, 400, 600, and 800 mM) for 7 days. Representative photographs show the concentration-dependent effects of salt stress on plant growth and morphology.

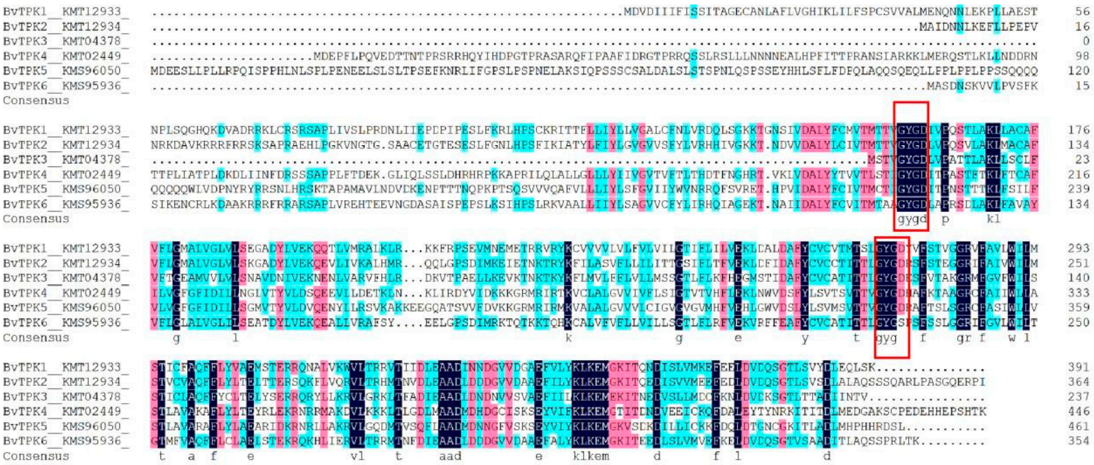

Figure S2. Multiple sequence alignment of BvTPK proteins. Multiple sequence alignment of six BvTPK proteins (BvTPK1–BvTPK6) was performed using DNAMAN. Conserved amino acid residues are highlighted according to sequence similarity. The conserved pore-forming region and the characteristic GYGD selectivity filter motif of TPK/KCO family proteins are indicated by red boxes. The alignment revealed conserved sequence features among BvTPK proteins, supporting their classification as members of the TPK/KCO family.
