# Supplementary material for: Genome-Wide Identification of the Two-Pore Potassium Channel Gene Family in Sugar Beet (Beta vulgaris L.) and Functional Analysis of BvTPK2 in Salt Tolerance
Source: Plants (Basel). 2026 Oct 15;15(20):3156. doi: 10.3390/plants15203156 (PMC13709052; doi:10.3390/plants15203156)
Supplement: Supplementary file 1 [file plants-15-03156-s001.zip › Supplementary File S1 TPK_KCO Protein Sequences.pdf]

>BvTPK2 (KMT12934)
MAIDNNLKEFLLPEPVNRKDAVKRRRFRRSKSAPRAEHLPGKVNGTGSAACETGTESESLFGNLHPSFIKIATYLF
IYLGVGVVSFYLVRHHIVGKKTNDVVDALYLCIVTMTTVGYGDLVPQSVLAKLMACAFVFLGMALVGLVLSKGA
DYLVEKQEVLIVKALHMRQQLGPSDIMKEIETNKTRYKFILASVFLLILITTGSIFLTFVEKLDFIDAFYCVCCTITTLG
YGDRSFSTEGGRIFAIVWILMSTVCVAQFFLYLTELTTERSQKFLVQRVLTRHMTNVDLEAADLDDDGVVDAAEF
ILYKLKEMGKITQEDISVVMEEFEDLDVDQSGTLSVSDLALAQSSSQARLPASGQERPI
>BvTPK1 (KMT12933)
MDVDIIIFISSITAGECANLAFLVGHIKLILFSPCSVVALMENQNNLEKPLLAESTNPLSQGHQKDVADRRKLCRSR
SAPLIVSLPRDNLIIEPDPIPESLFKRLHPSCKRITTFLLIYLLVGALCFNLVRDQLSGKKTGNSIVDALYFCMVTMTT
VGYGDIVPQSTLAKLLACAFVFLGMALVGLVLSEGADYLVEKQQTLVMRALKLRKKFRPSEVMNEMETRRVRYK
CVVVVLVLFVLVILGTIFLILVEKLDALDAFYCVCVTMTSLGYGDYVFSTVGGRVFAVLWILMSTICFAQFFLYVAE
MSTERRQNALVKWVLTRRVTIIDLEAADINNDGVVDGAEFVLYKLKEMGKITQNDISLVMKEFEELDVDQSGTL
SVYDLEQLSK
>BvTPK3 (KMT04378)
MSTVGYGDLVPATTLAKLLSCLFVFTGEAMVVLVLSNAVDNIVEKNENLVARVFHLRDKVTPAELLKEVKTNKTK
YKFLMVLFFLVLLMSSGTLFLKFHEGMSTIDAFYCVCATITTLGYGDESFVTAKGRMFGVFWILSSTICLAQFFYCL
TELYSERRQRYLLKRVLGRKLTFADIEAADLDNDNVVSVAEFIILKLKEMEKITNEDVSLLMDCFKNLDVDKSGTLT
TADIINTV
>BvTPK4 (KMT02449)
MDEPFLPQVEDTTNTPRSRRHQYIHDPGTPRASARQFIPAAFIDRGTPRRQSSLRSLLLNNNNEALHPFITTPRA
NSIARKKLMERQSTLKLLNDDRNTTPLIATPLDKDLIINFDRSSSAPPLFTDEKGLIQLSSLDHRHRPKKAPRILQLA
LLGLLLYIIVGVTVFTLTHDTFNGHRTVKLVDALYYTVVTLSTIGYGDITPASTFTKLFTCAFILVGFGFIDILLNGLVTY
VLDSQEEVLLDETKLNKLIRDYVIDKKKGRMRIRTKVCLALGVVIVFLSIGTVTVHFLEKLNWVDSFYLSVTSVTTV
GYGDHAFKTAAGRCFAIIWLLASTLAVAKAFLYLTEYRLEKRNRRMAKDVLKKKLTLGDLMAADMDHDGCISKS
EYVIFKLKEMGTITDNDVEEICKQFDALEYTYNRKITITDLMEDGAKSCPEDEHHEPSHTKT
>BvTPK5 (KMS96050)
MDEESLLPLLRPQISPPHLNLSPLPENEELSLSLTPSEFKNRLIFGPSLPSPNELAKSIQPSSSCSALDALSLSTSPNL
QSPSSEYHHLSFLFDPQLAQQSQEQLLPPLPPLPPSSQQQQQQQQQWLVDPNYRYRRSNLHRSKTAPAMAVL
NDVDKENPTTTNQPKPTSQSVVVQAFVLLLIYLSFGVIIYWVNRRQFSVRETHPVIDALYFCIVTMCTIGYGDITP
NSTTTKLFSILFVLVGFGFIDILLSGMVTYVLDVQENYLLRSVKAKKEEGQATSVVFDVKKGRMRIRMKVALALGV
VVLCIGVGVGVMHFVEHLGWVDSLYLSVMSVTTVGYGDRAFTSLSGRCFASIWLLVSTLAVARAFLYLAEARIDK
RNRLLAKRVLGQDMTVSQFLAADMDNNGFVSKSEYVIYKLKEMGKVSDKDILLICKKFDQLDTGNCGKITLADL
MHPHHRDSL
>BvTPK6 (KMS95936)
MASDNSKVVLPVSFKSIKENCRLKDAAKRRRFRRARSAPLVREHTEEVNGDASAISPEPSLKSIHPSLRKVAALLII
YLSAGVVCFYLIRHQIAGEKTNAIIDALYFCVITMTAAGYGDLAPRSDLAKLFAVAYVFLGLALVGLILSEATDYLVEK
QEALLVRAFSYEELGPSDIMRKTQTKKTQHKCALVFVFLLVILLSGTLFLRFVEKVRFFEAFYCVCATITTLGYGSPSF
SSLGGRIFGVLWILTGTMFVAQFFLCLAELSTEKRQKHLIERVLTRRMTNFDIEAADLDDDGVVDAAEFALYKLKE
MGKITEEDLSLVMVEFKELDVDQSGTVSAADITLAQSSPRLTK

>AtTPK1 (AT5G55630)
mssdaartpllptekidtmaqdfnlnsrtsssrkrrlrrsrsaprgdcmynddvkidepppphpskipmfsdlnpnlrrvi

mflalyltigtlcfylvrdqisghktsgvvdalyfcivtmttvgygdlvpnssasrllacafvfsgmvlvghllsraady
lvekqeallvrafhlrqsfgptdilkelhtnklrykcyatclvlvvlfivgtiflvmvekmpvisafycvcstvttlgyg
dksfnseagrlfavfwiltssiclaqfflyvaelntenkqralvkwvltrritnndleaadldedgvvgaaefivyklke
mgkidekdisgimdefeqldydesgtlttsdivlaqttsqiqr

>AtTPK2 (AT5G46370)
mandgngdnnddplrqylmnprinppppslltlpenndvtipmpitplelknrlifgsfvrsrkesslpidalsqnpsts
ssattsfsdstdlllpltepnkpvrkskptinfhrsktapamaainnishpndpktdqqsdsktivnqavallvvylslg
vliywlnrdsynvkqthpvvdalyfcivtmctigygditpdsvvtklfsiffvlvgfgfmdillsgmvtyvldlqenyml
etarneslnlndrdkvrsyiidvkkgrmrirlkvglalgvvvlclgfgvlimhfvekigwldsfyfsvmsvttvgygdra
fntlagrllaamwllvstlavarailflaesrvdkrnrerakkvlgesmsisqfldadidcngcvskaefviyklkkmdk
itekdinpigfqfdkldrtnsgritlldllesstkdlptatsi

>AtKCO3 (AT5G46360)
mpmtpsefknrllfgslprsssdptdlqftepnvppslfslpehnddtatdmapdqeteqsvsksiarqalallvvylsl
gvliywltldsdnayqthpvavalyffvvtfcgflivhfvvkigwldsfcfsvmmvttvgfgdrafntwlgtflaavwll
vstlavaraflfladaradkrnrerakkvlgesisisqffaadidndgrlslaefaiyklkqmekitqedfiqicnhnth
ynmiedgsgksavldivepv

>AtTPK4 (AT1G02510)
meeenllnenllhpnesspeetqvttvskskwtilvlamilllvyltfgvctysffrdqfsgtetnlfvdafyfsivtfs
tvgygdivpststtkiltivlvstgvvfldyllnrvvshvlslqenaildrinktrnrairdhiaedgkirlkwklclaf
cavglcvgsgalflhvferldwldsvylsvisvttvgygdktfktvegrgfavfwlllstiamatlflylaemridrttv
mklppsesefivfklresgriseddikqivrefenleevpssgs

>AtTPK5 (AT4G01840)
meplispqprfrlqpipenpssssssasitiprsisntsffheisqerlllhhqdleqsvqddkedqdsdsdetnrflsq
trplhrsrtapamviikdlrtkppetkkpspvsksiirqaifllivyltlgvsiysfnrdhysgiethpvvdalyfcivt
mctigygdiapltpwtkifavvfvlfgfgfldillsgvvnyvldlqesmiltgiqtrqhhqhhhhhrfsakdyiidfekg
rmrirmkvclalcvvvlcigvgalvlhfveelgfvdsvylsvmsvttvgygdrafktlqgrlfaavwllvstlavarafl
ylaearidrrhrkavklalnreitvddllkadtyqhgfiskseyivlklkemgkitqkdidqvviqfekldpnqigkitl
pdllgdpl

>AtTPK3 (AT4G18160)
manegsdpllqymisprlkkppqllfplpednevaipmpmtpsefkerlifgpfscsprdsshfidsmkqpspsssstav
nnpfsdsstldpllpppppqpepwlsdqtsshcqghalhrsktapamavindlhhpirqkdptetsrsvvrqafallvvy
lslgvliywlnrdhyvvnqthpvvdglyfcivtmctigygditpnsvvtklfsimfvlvgfgfidillsgmvsyvldlqe
symldsakrrdepekrrsyiidvkkgrmrirlkvalalgvvvlciavgvgimhfieeigwldsfylsvmsvttvgygdra
fktlpgrlfaaiwllvstlavaraflylaearvdkrnrerakkvlcetmsvsqffaadidnngcvskaeyviyklkemek
itdkdilpiskqfdkldrcsngkitlldlleggsgd

>GmKCO2-1 Glyma.02G285700.1
MQESLLRSCSRKADSPNRSSPGELSIHDLALALAPSLFQSSSHHVTNEAKTSMHLIANLAFKKGKFIHRSRSAPSL
LFTD
MGLDFQEPSEPHKSSTSIVRLSFLGVFLYVAIGVTVYMTSGSFRGTTTFRPVDAVYFTMVTLCTIGYGDIVPDSTF
TKIF
TCGFILVGFGFLGFLLNGLVAYICDTQEAFLLSMMDENRYKKILRTYMVDEEKGRMRIRTKVCLALAVVIGCIAIG
TVTV
HLVEDLNWDDSIYLSITSVTTVGYGDFSLRTVTGRCFAIIWLLVSTLAVARAFIYLTEYSIHKRNRKMAQWVLQKKI
TLS
DLAAADLDNDGSISKSDFVIYKLKQMGKITEIDILQISKQFDSLEHGMYGKITLADLMDTV
>GmKCO1-1 Glyma.02G299700.1
MANNGAKDPLLSGSLDATQKTKQQLLNKRRLLRSRSAPHAELVPTETNCNESIPRTASIFQNLHPSFKRMAIYLA
VYLGV
GALIFYLVRNQIKGQKTDGILDALYFTIVTMTTVGYGDLVPNSHLAKLLACAFVFSGMALIGLIVSKAADYLVEKQE
LLL
VKAMRMHQKIGSTEILREVETNKTRYKLFLVFSLLLILIIVGTIFLVTVEKLDVIDAFYCVCSTITTLGYGDQSFSTQA
G
RIFAVFWILTGTITLAQLFVYIAELNTEIRQKELVKWVLTRKVTNLDLEAADLDEDGTVGAAEFVIYKLKEMGKISQ
EDI
SLVMQEFEQLDVDDSGTLSTSDITLAQSS
>GmKCO3-1 Glyma.03G223900.1
MEDEPFLLTTTTSQPITDFLLPSSKSFNDVTTTTRARDAAAQEIQSEQQQPPKKKKLSRCKTAPAMVTMRDLKP
KTPQLP
KPQTSSIIRQGIWLLAMYLSIGVAIYSFNRDRFSGIETHPVVDALYFCIVTMCTIGYGDIAPLTPFTKIFACAFVLVGF
G
FIDILLSGLVNFVLDLQENMILTGLQMGASDQREGFSARNYIVDVAKGRMRIRLKVGLALGVVVLCIGIGGLVLYF
VEGL
DWVDSIYLSVMSVTTVGYGDRAFKTLPGRLFAAIWLLFSTLMVARAFLYLAEARIDRRHRRMAKKVLHREITVE
DLLAAD
INNTGFISKSEYVIFKLKEMGKIQEKDVLQICDQFRKLDPSNCGKITLPNLLGGSL
>GmKCO3-2 Glyma.05G127500.1
MEKEPLLPYFSPRKKPQLCPLPEHDEIVLPMTPSEFKDRLIFGPSPSSASPRDPSPLADALTLSHNSPKCSSSNSQD
YAS
SPLFDSQQKQQQLNSWLLDPNYESWRKTNLHRSKTAPAMAVISDFNPHSTVQRPKFASQSIVCQGVILLALYLA
LGVVIY
WFNRHNFTATETHPIVDALYFCIVTMCTIGYGDITPNSTATKLFSILFVLVGFGFIDILLSGMVSYVLDLQENHMLT
AVK
GRRGEKDGKSYIIDVKKGRMRIRLKVALALGVVVICIGVGVGVMHFVEKLGWLDSFYLFDHAFKTMHGRIFAAI
WLLVST
LAVARAFLYLAEARVDKRHRRMAKWILGQDMTVSEFLAADIDNNGFVSKSEYVIYKLKEMGKVSEKDIMQVSE
KFDRLDA
GNCGKITLADLMGNHN
>GmKCO3-3 Glyma.08G082200.1

MEKEPLLPYFSPRKKPQPFPPLCPLPEHDEIVLPMTPSEFKDRLIFGPSPSSASPRDPSPLADALTLSYNSPKCPSSS

NS

QDYASPPLFDSQQQQPLNSWLLDPNYESWRKTNLHRSKTAPAMAVISDFNPHTAVQRPQFASQSIVRQGVILL

ALYLALG

VVIYWFNRHNFTATETHPVVDALYFCIVTMCTIGYGDITPNSTATKLFSILFVLVGFGFIDILLSGMVSYVLDLQEN

HML

TAVKGRRGEKDGKSYIIDVKKGRMRIRLKVALALGVVVICTGVGVGVMHFVEKLGWLDSFYLSVMSVTTVGYG

DHAFKTM

HGRIFAAIWLLVSTLAVARAFLYLAEARVDKRHRRMAKWILGQDMTVSEFLAADIDNNGFVRSSLHLGIGGQDL

TISKSE

YVIYKLKEMGKVSEKDIMQVSEKFDRLDAGNCGKITLADLMENHN

>GmKCO1-2 Glyma.14G014100.1

MINYGEARSYGITCIESQAFRSLCHTHISSPSATTTNHFKEMDAFSAFSLNEGTQLRRSIWLVSVVSDDPNDFILHL

WWS

YIADPCFLNSSAVGKGAYMANNGVKEPLLWVSLDATQKTKQQLNKRSLLRFRSAPHAEIVPTETNGNESIPHSA

SIFQNL

HPSFKRVAIYLAVYLGVGALIFYLVRNQIKGQKTDGILDALYFTIVTMTTVGYGDLVPNSHLTKLLACAFVFSGMAL

IGL

IVSKAADYLVEKQELLLVKAMRMHQKVGSTEILREVQTNKTRYKLFLVFFLLLILIIAGTIFLVTVEKLDVIDAFYCVC

S

TITTLGYGDQSFSTQAGRIFAVFWILTGTITLAQLFLYIAELNTEIRQKELVKWVLTRKVTNSDLEAADLDVDGTVR

AAE

FVIYKLKEMGKISQEDISLVMQEFEQLDVDDSGTLSTSDITLAQSS

>GmKCO2-2 Glyma.14G028700.1

MFMNRRNHEDLHLFTIILNQEIRRLSTQVIRVKWSLHAREEIVFELLQQLRGNAAIVLLEGVMKSTRQMIEEQE

AVRGKK

QTHQTDSSHHVTNEAKTSMHLIANLAFNKGKIILRSCSAPSLLFTDTGVDFQEPSEPHKSSTSIVKLSFLGVFLYVA

TGV

TVYMTSGSFRGSTTFRPVDAVYFTMVTLCNIGYVDIVPDSTFTKIFTCAFILVGFGFLGFLLNGLVAYICDIQEAFLL

SM

VDENRYKKILRTYMVDEEKGRMRIRTKFCLALAVVIDCIAIGTVTVHLVEDLNWDDSIYLSITSVTTVGYGDFSLR

TVTG

RCFAIIWLLVSTPAVARASIYLTEYSIQKRNCKMAQWVLQKKITLSDLAAADLDNDGSISKSDFVIYKL

>GmKCO1-3 Glyma.17G006800.1

MGGDESRESLLSEARDHSHLNEISDLQRRRPRRGKSSDKENNLKEKNVVQNPLHSQYIDPKQEAEFHFKTVLL

WLAAYLG

GGTLCFLLTSHQIKGIKTNGFLDAIYFCVVTMTTVGYGDLVPDSQLAKLLACIYVFTGMALVGLILSKAADYIVEKQ

EIF

LVRTLFKGENFGPEELSKEVETNKAKYKFILAASVFLVLMISGTIFLHYIENLDFVDALYCVCSTVTTLGYGDKSFSTT

I

GRAFAVFWILSSTICLAQSFAYLAEFYTEERQKAMAKIVLTRKLSLLDLEAADLDGDHVVSATEFVLYKLKEMGKIN

QED

ILVVMDIFRKLDVDKSGTLTEADLKYSESSKP

>GmKCO3-4 Glyma.19G221000.1

MFSFLLLFSAMEDEPFLTTTSQPIADCLLPSSKSFNDVTTTRARDVAAAAAEEIQSEQQQPPKKKKLSRCKTAPA
MVTMR
DLKPKTPQLPKPQSSSIIRQGMWLLAVYLSIGVVIYSFNRDRFSGIETHPVVDALYFCIVTMCTIGYGDIAPLTPFT
KIF
ACAFVLVGFGFIDILLSGLVNFVLDLQENMILTGLQMGASEREGFSARNYIVDVAKGRMRIRLKVGLALGVVVM
CIGIGS
LVLYFVEGLDWVDSIYLSVMSVTTVGYGDRAFKTLPGRLFAAIWLLFSTLMVARAFLYLAEARIDRRHRRMAKKV
LHREI
TVQDLLAADINNTGFISKSEYVIFMLKEMGKIQEKDVLQICDQFRKLDPSNCGKITLPHLLGGSL

>Q850M0 OsTPKa
MDDNSIQQSLLADNPNVLQRKPSEGVNRFRRCRSTPSTDPLQGPPEKGSSVKAKELFKEM
RPSFRLVGLLLFIYLLVGVLAFYAVMDEISGKRTNRVLDALYFCVVTMTTVGYGDLVPNN
DTTKLLACAFVFMGMAVVALFVSKVADYLVEKQEVLFFKALHTNLKGGETKMLRAIETNR
IKYKFYTNALLLVLSIISGTVFLWKVEKLSLVDSFYCVCATITTLGYGDKSFSSKLGRVF
AVFWIITSTIIMAQFFMYLAEIYTERRQKMLANWVLTRKMTKMDLEAADLDDDRQVGAAE
FVVYKLKELGKINQEEISSFLEEFEKLDVDHSGTLSPYDLTLAQSAQ

>Q8LIN5 OsTPKb
MAALDQQPLLHDGGDQKPPPEGAARRFRRCRTAPSSEPPPTDKDNSSAADAPPKTLFTGG
GRPSFRLVGLLLVAYLLLGTIAFYLAMDHMSGTRTTRALDALYFCVVTMTTVGYGDLVPA
SDAAKLLACAFVFAGVAVVGTFLSKAADYLVEKQEALLFRALHSHTMVRAMEMNKVRYKL
YTAGLLLVAAVASGTVVLWKVEGMRAVDAFYCVCATVTTLGYGDRSFSSEGGRAFAVAWI
TVSTVVVALFFLYAAELYTERRQRELARWVLRRRTTNMDLEAADLDGDHRVGAADFVLYK
LKELGKISQEDISEFLDEFDNLDADHSGTLSPADLAAAQPTPDPPPSLR

>Q69TN4 OsTPKc
MDTEPLLSPLSPSPHLLHPLPEHAEVSTFSPPLSPCPSPASSYKERIIFGAHPPPPPPPP
PPPPPPPRGRRYYRRVSGDDLDVPSCSSSPSPPSDEENPPPNPPSLFDFIGGRTNLHRSR
TAPAMAPLNAAAIAAAAASGDSRNPPPPPRRPAIVLHAFLFLLAYLAMGVTFYAALPGNF
TSSAGPTHPVADALYFCIVTLCTIGYGDITPATPAAKLFSISFVLIGFGFVDILLSGMVS
YVLDLQEHLLITALKNPRSVRKHRHNYIFDLKKGRMRVRMKVALALTVVAICVGVGAAVL
KRVENLGWLDAVYLAVMSVTTVGYGDHAFRTLAGRLFASAWLLVSTLAVARAFLYLAEMR
IDKRHRAMANWVLSRDMTVSEFLAADIDNNGYVTKSEFVVYKLKEMGKISEKDIMMICDQ
FQRMDSGNCGKITLSDLLESHQLVTDLNEKKKGKKS
