# Supplementary material for: Genome-Wide Identification of the Two-Pore Potassium Channel Gene Family in Sugar Beet (Beta vulgaris L.) and Functional Analysis of BvTPK2 in Salt Tolerance
Source: Plants (Basel). 2026 Oct 15;15(20):3156. doi: 10.3390/plants15203156 (PMC13709052; doi:10.3390/plants15203156)
Supplement: Supplementary file 1 [file plants-15-03156-s001.zip › Supplementary Table S1 Primers.pdf]

Table S1 Primers used for qRT-PCR

**Primes**	Forward(5'-3')	Reverse(5'-3')
BvTPK1	TGAGCACTGAAAGGAGGCAA	ATACAAACTCAGCGCCGTCT
BvTPK2	TCTCAACAGAGGGAGGTCGT	TATGCCGAGTTAGGACACGC
BvTPK3	CGTCGATAGAGCGAGAGGTG	ATTCCTTGCTGCCTTCACCA
BvTPK4	CCACGGCGTCAATCATCTCT	GCAATGGAATTAGCTCGCGG
BvTPK5	AGGTCTAAAACTGCCCCAGC	GTGGGCTTAGGCTGATTGGT
BvTPK6	CCGTCTTAAGGATGCTGCGA	CTTATTGCGGAAGCATCGCC
BvActin	AGATGGTCGATGAGGTTCGC	GCCGAGATTCACCTGGACAT
BvTPK2-GFP	AAGTCCGGAGCTAGCTCTAGatggccatcgacaacaacttgaaggag	AGCGGCCGCTGTACAGGATCttcaataggtctctcctgaccagaagc
Kan	TTGTCACTGAAGCGGGAAGG	CGGCGATACCGTAAAGCAC

Note: The Kan primer pair was used to verify transgenic-positive plants by PCR amplification of the kanamycin-resistance marker gene.
